# Intraspecific Variation in Dorsal Colour Patterns of *Amphibolurus muricatus* Lizards From the Perspective of Relevant Observers

**DOI:** 10.1002/ece3.71944

**Published:** 2025-08-11

**Authors:** Jonathan W. Salisbury, Richard A. Peters

**Affiliations:** ^1^ Animal Behaviour Group La Trobe University Melbourne Victoria Australia; ^2^ Department of Ecological, Plant and Animal Sciences, School of Agriculture, Biomedicine & Environment La Trobe University Melbourne Victoria Australia

## Abstract

The visual environment animals occupy is often comprised of spatially distinct microhabitats featuring different and varying backgrounds, lighting conditions, temperatures, feeding opportunities and threats. For species employing colouration in some capacity, efficacy can vary substantially between these; thus, we expect some variation within species to adapt to the traits of specific microhabitats. Cryptic animals can be particularly sensitive to changes or choices in habitat and predation threats and often display intraspecific variation of their colour patterns. One such species, the Jacky dragon ( *Amphibolurus muricatus* ), occurs across a wide geographic range incorporating several distinct habitat types and possesses dorsal camouflage patterns that aid in avoiding detection from a range of potential predators. Here we investigate intraspecific variation of the dorsal patterns of *A. muricatus* within the context of relevant observers at multiple viewing distances. Using multispectral photography, contemporary analysis software and specifically constructed visual models for the Jacky dragon and two avian predators, we quantified patterns of individuals collected across the geographic range of the species. Larger lizards appear to have more distinct dorsal patches to lizards at close range, but avian predators see reduced pattern diversity and complexity. At a distance, both predators see fewer clusters, and size has less effect. Close‐up, males show more distinct patches, contrast and luminance to lizards, but lower contrast to kookaburras. Furthermore, differences are also apparent between genetic clades and habitat characteristics, with lizards in coastal heath exhibiting greater pattern diversity and complexity to all observers. Importantly, these differences are mediated by observer identity, as significant effects are not consistent across different visual systems. Our results suggest that variation in the visual appearance of these lizards is multifaceted and in response to both general and local selection pressures.

## Graphical abstract

This study examined dorsal pattern variations across the geographic range of an Australian lizard using multispectral photography and visual models for lizards and avian predators. Findings reveal differences in dorsal colouration based on sex, body size, genetic clades and habitat type. However, these variations are perceived differently depending on the observer's visual system. The study highlights that the species' colouration is influenced by both broad and localised selection pressures, suggesting a complex interplay between environmental adaptation and predator vision in shaping their camouflage strategies.

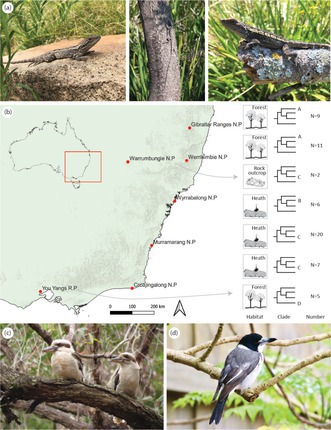

## 1 Introduction

Animal patterns—such as colouration, striping, spotting and other visual markings—exhibit significant variation within species (Stuart‐Fox et al. 2021; Cuthill et al. 2017), often changing across different parts of a species' distribution (Aguilar et al. 2024). This can be manifested as discrete polymorphisms (Teasdale et al. 2013) or continuous variation (Weiss 2006). Such intraspecific variation is shaped by multiple, interrelated evolutionary processes. Phylogenetic influences reflect the genetic legacy of evolutionary ancestry, shaping the range of pattern types a species can express through inherited developmental pathways and constraints (Olsson et al. 2013; Wellenreuther et al. 2014). Natural selection plays a crucial role as well, favouring coloration and patterns that enhance survival in specific environments, with particularl patterns favoured to match local conditions or local predator communities (Rojas and Endler 2013; Kang et al. 2015; Smithers et al. 2018; Lovell et al. 2013; Barnett et al. 2021; Tong et al. 2019; João et al. 2023). Additionally, morphological traits such as body size and shape can interact with patterning, influencing functions like thermoregulation or visibility (Miñano et al. 2024). Sexual dimorphism in patterning is another important source of variation, with males and females exhibiting distinct patterns driven by sexual selection, signalling fitness or social status (Maan and Cummings 2009; White et al. 2012; Olsson 1994). Studies on lizards have been especially valuable in this field, offering insights into how the diversity of animal patterns within a species reflects a dynamic balance between inherited constraints, environmental pressures and reproductive strategies (Stuart‐Fox et al. 2021; Sanclemente and McBrayer 2025).

Discrete and stable polymorphs within a species are common examples used to illustrate genetic variation of traits (Stuart‐Fox et al. 2021; Sun et al. 2024). Polymorphism is driven by one or more genes and is often associated with significant variations in strategy between morphs (Kaliontzopoulou et al. 2007). For example, Tawny dragons ( *Ctenophorus decresii* ) show four distinct throat colour morphs in males (Teasdale et al. 2013), governed by a group of identified loci and alleles (Rankin et al. 2016). Interestingly, evidence suggests these morphs are associated with differing conspecific interaction strategies, with varying levels of aggression per morph (Yewers et al. 2016). Certainly, colour variation is linked with genetic differences in polymorphic species (Jian et al. 2025; Sun et al. 2024; Lu et al. 2024), but phylogenetic influences arising from genetic differences are also observed in many species that do not necessarily exhibit discrete morphs (Brock et al. 2022; Storniolo et al. 2021; Dadda et al. 2023). In the widely distributed *Podarcis muralis* lizards of Europe, dorsal patterns are closely linked to their evolutionary history. Dadda et al. (2023) reported that the distribution of different patterns is strongly linked with random evolutionary forces like genetic drift. Furthermore, Storniolo et al. (2021) suggest phylogenetic differences might have contributed to population differences in appearance between populations of *Podarcis siculus* , which occur in different clades (subspecies). Taken together, the evolutionary history of focal species must be considered in attempts to explain pattern variation across geographic ranges of species.

Localised conditions represent a significant source of selective pressure on animal patterns and colouration (Sun et al. 2024; Lu et al. 2024; Sau et al. 2023), to the extent that many species show intraspecific variation in their colour and pattern traits to better suit the local microhabitat (Marshall et al. 2015; Kirchhof et al. 2012; Tong et al. 2019). For species utilising concealment‐orientated camouflage strategies such as background matching, pattern and colour variation likely reflect vegetation structure, substrate availability, weather and lighting conditions because they directly affect the efficacy of these strategies (Stevens and Ruxton 2019; Cuthill et al. 2017). The presence, structure and intensity of such environmental traits will often vary between habitats, locations, or populations and therefore the ideal phenotype will likewise vary (Merilaita et al. 2017). Indeed, the significant chromatic variations evident in the genus Podarcis can be attributed to species‐specific habitat preferences (Escoriza 2024), while in the spiny‐footed lizard (*Acanthodactylus erythrurus*), dorsum luminosity was positively related to ground luminosity upon which the lizards are observed (Cuervo et al. 2024). Phenotype‐environment matching is relevant because predation represents a significant selective agent on the phenotypes of camouflage (Sanclemente and McBrayer 2025). The ability of predators to detect and recognise camouflaged individuals is a strong selective force, as for many species being detected can directly translate to the need for costly escape behaviour, injury, or death (Cuthill 2019). Predation is consistently linked to variations in local camouflage phenotypes directly or indirectly by selecting for better matching to the available habitat (Sanclemente and McBrayer 2025). As natural selection mediates phenotype‐environment matching, variation in colour and patterns might vary in response to predator type and abundance at different locations, as well as the structure of the local microhabitat.

Colour and pattern often differ between males and females of a species (Stuart‐Fox and Ord 2004). These differences might reflect distinct ecological roles or behavioural strategies between the sexes, influenced by factors like predation risk, habitat use and reproductive investment. Cuervo et al. (2024) describe sex differences in the appearance of the dorsum of spiny‐footed lizards and nominate males to be more cryptic than females, pointing to differences in activity patterns between the sexes as a likely explanation. However, sexual dimorphism of this kind is also considered to be driven by sexual selection and arises from the selective pressure of mate choice (Edward and Chapman 2011; Stuart‐Fox and Ord 2004) or competition (Slatkin 1984). As in other taxonomic groups, male lacertid lizards tend to exhibit more complex and diverse colourations than females (Pérez i de Lanuza et al. 2013), which might be a consequence of sexual selection operating through male–male competition (Olsson and Madsen 1998; Ord et al. 2001). Madeiran wall lizard (*Teira dugesii*) males exhibit higher overall hue than females, which might reflect the result of sexual selection in favour of more colourful males (Aguilar et al. 2024). However, luminance values were higher in female Madeiran wall lizards, with Aguilar et al. (2024) speculating about the role of hormones in dictating differences in appearance between sexes (and between seasons). While hormonal control of body colour in spiny‐footed lizards is supported (Fresnillo et al. 2019), Jian et al. (2025) rule out body condition as an explanation for sex differences between male and female Stejneger's grass lizards (*Takydromus stejnegeri*). The effect of body condition warrants further investigation, as do more basic differences due to variation in body size (Jian et al. 2025). Indeed, Aguilar et al. (2024) suggest that colour expression can be linked to ontogenetic effects because of pigment accumulation and structural changes in skin surfaces as lizards grow.

Empirical investigation of the variation of animal colouration has typically considered phenotypes objectively, and more recently these investigations have benefited from quantitative tools to consider appearance from the perspectives of stakeholder species and the associated limitations of relevant species' visual systems (van den Berg et al. 2020). Pattern features or variations observed by humans may fall outside the region of detectable difference for relevant observers and, in effect, be irrelevant random noise to the biological actors (Troscianko and Stevens 2015; Merilaita et al. 2017). Therefore, variation should be considered within the ecologically relevant context, and this includes the visual ability of observers, which limits sensitivity and information uptake (Stevens and Merilaita 2009; Stevens and Ruxton 2019). This includes utilising the cone sensitivity profiles of target observers, which can vary substantially between species in terms of number and peak sensitivity and influence the perception of colour and patterns (Osorio and Vorobyev 2005). In addition, the ability to resolve pattern detail is influenced by the species' spatial acuity, which changes with viewing distance (Caves and Johnsen 2017), causing the detail, contrast and complexity of a visual pattern to vary as an observer moves away. Colour and pattern should therefore be quantified with respect to the sensory capabilities of intended observers and their position relative to the source. There are a variety of open‐source analytical tools that facilitate such investigations, including the quantitative colour pattern analysis (QCPA) framework (van den Berg et al. 2020) that integrates calibrated digital photography (Stevens et al. 2007), visual modelling of species perceptual capabilities and pattern analysis within the multispectral image calibration and analysis toolbox (micaToolbox; Troscianko and Stevens 2015). QCPA supports advanced analyses like colour adjacency, visual contrast and boundary strength, enhanced by tools such as receptor noise limited (RNL) clustering and local edge intensity analysis (LEIA) for more accurate, biologically relevant assessments. This flexible system allows researchers to explore various ecological and evolutionary topics through detailed visual data analysis.

**FIGURE 1:**
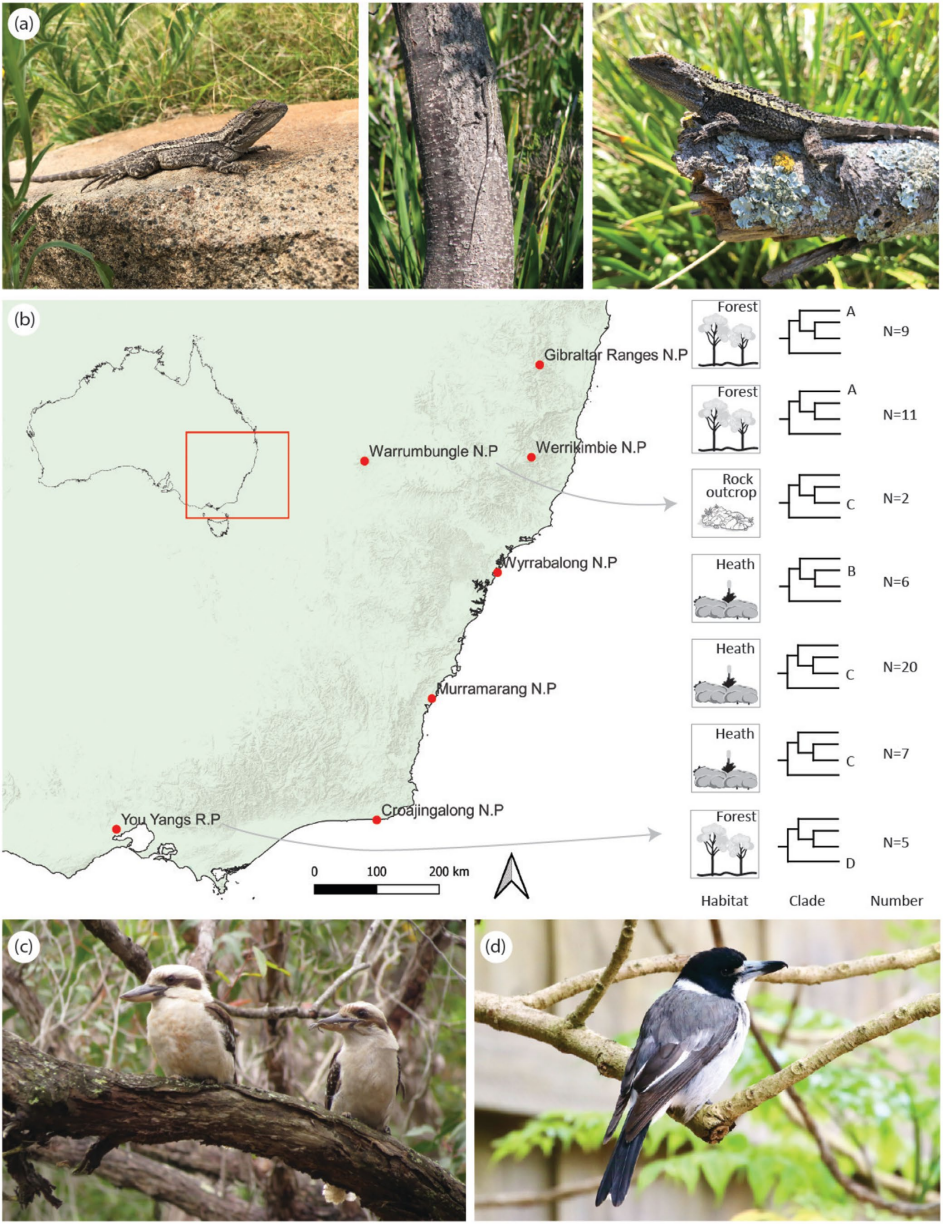
(a) Images of *Amphibolurus muricatus* in natural settings and (b) locations where lizards were captured and photographed in south‐eastern Australia, indicating the habitat type, genetic clade and sample size for each site. (c) The laughing kookaburra (*Dacelo novaeguineae*) and (d) grey butcherbird ( *Cracticus torquatus* ) were used as representative predators.

Jacky dragons ( *Amphibolurus muricatus* ; Figure 1a) are small (70–120 mm snout‐vent length) semi‐arboreal agamid lizards, common along some inland and most coastal areas of south‐eastern Australia and occurring in a range of habitat types, including coastal heathland and open dry forest (Figure 1b). Their energetic signalling and communicative displays have attracted significant research effort because of trait variation in response to different environments (Peters and Evans 2003; Peters et al. 2007; Barquero et al. 2015; Ramos and Peters 2017), and recent genetic analyses show five distinct clades separated by geographic features (Pepper et al. 2014). Jacky dragons possess dorsal patterning dominated by two dorsal‐lateral stripes with substantial variation in internal pattern configuration. These lizards exhibit the characteristic ability to darken and modify contrast within pattern features that many agamid lizards employ, presumably for thermoregulation (Cadena et al. 2018) and likely crypsis (Smith et al. 2016) and communication (Dickerson et al. 2020), but otherwise cannot rapidly modify their patterning. They are often found basking on fallen branches or rocks, though captive experiments suggest that basking sites are not chosen at random, with favourably patterned substrates and the presence of nearby vegetation cover being important (Salisbury and Peters 2019). Avian species that rely on visual detection are the most significant predation threat for adult Jacky dragons and include the laughing kookaburra (*Dacelo novaeguineae*; Figure 1c; Allen et al. 2009) and grey butcherbirds ( *Cracticus torquatus* ; Figure 1d) and nankeen kestrels ( *Falco cenchroides* ) (Stuart‐Fox et al. 2003; Sazima 2023). The aim of this study was therefore to quantify the relative contribution of phylogenetic relatedness (clade), sex, size and habitat type to the dorsal patterns of these lizards. We extend previous work by examining the spatial and spectral aspects of pattern by modelling the visual capabilities of multiple relevant observers and anticipate the relative contribution of these factors will be mediated by observer type due to the differences in visual capabilities of lizards and avian predators.

## 2 Methods

### 2.1 Study Animals

Sixty individuals were located and photographed at seven sites across Victoria (VIC) and New South Wales (NSW), Australia (Figure 1b): Croajingalong National Park (VIC), You Yangs Regional Park (VIC), Murramarang National Park (NSW), Werrikimbe National Park (NSW), Wyrrabalong National Park (NSW), Gibraltar Ranges National Park (NSW) and Warrumbungles National Park (NSW). These sites were selected to sample different genetic clades and habitat types and were accessed across several expeditions between 2017 and 2020. Lizards were located and captured during the breeding season (October–February) in 2017–2018, 2018–2019 and 2019–2020. They were photographed immediately to minimise any time to darken their colouration, assessed for sex and snout‐vent length (SVL), and returned to the perch upon which they were first located, all within approximately 5 min of capture. Lizards found to be gravid or at any stage of shedding were not photographed.

### 2.2 Image Acquisition

#### 2.2.1 Camera Equipment and Calibration

Images of the dorsal patterns of lizards were captured using a UV‐sensitive digital SLR camera. A Canon 7D camera (Canon Inc.) was modified to be sensitive to light in the UV and infrared (IR) ranges by Camera Clinic (Collingwood, Melbourne, Australia). The original sensor filter was replaced with non‐filtering material that transmits light in the range 280–1100 nm (whereas unmodified cameras are limited to 400–700 nm). Canon 7D cameras have been successfully used by others in this capacity (e.g., Yeager and Barnett 2020). We attached a Nikkor EL 80 mm f/5.6 lens (Nikon Pty Ltd) to the camera body, as it allows for sufficient transmission of UV light to at least 350 nm and variable transmittance between 350 and 310 nm. This lens is relatively affordable and frequently used by enthusiasts and studies of animal colouration alike (da Silva et al. 2020). The modified camera and lens no longer filter light from the sensor, and so a Baader U‐filter (2″) (Baader Planetarium, Mammendorf, Germany) was used for UV spectrum photography. This absorptive/dichroic UV‐pass filter cuts out visible and IR light while transmitting between 320 nm and 380 nm and has been employed by numerous studies investigating UV in some capacity (e.g., Marshall and Stevens 2014; Marshall et al. 2016; Gómez et al. 2018). A Baader UV/IR cut filter (2″) (Baader Planetarium, Mammendorf, Germany) was used for visible‐spectrum photography (300–700 nm), with no light bleed until very high into the IR spectrum (1120 nm).

The camera was calibrated in VIS and UV spectrums via a set of ‘Inscribe’ 64‐piece artist pastels, with the UV‐fluorescent pieces discarded. Using a JAZ spectrophotometer (Ocean Optics Inc.) and standard probe, each pastel's reflectance was measured outside under constant sunlight conditions. A calibration profile was then generated using multispectral photographs of the array in natural lighting conditions. Combining the known reflectance data of the colour standards with the information captured in photographs of the standards allows the micaToolbox package to estimate the camera/lens combinations' particular response to wavelengths across the spectrum for accurate colour data interpretation. A white standard (layered white PTFE tape, measured at 75% reflectance 300 nm–700 nm) was included in all photographs.

#### 2.2.2 Camera Settings

Photographs were captured in lossless RAW format to avoid any potential issues with compression or colour information loss. The camera's natural ISO rating of 200 was used as images were captured in natural daytime conditions when lighting was not a constraining factor. We used an aperture of f/8 to have more of the scene in focus and maintain a sharp image and held this constant for all photography, managing exposure by adjusting shutter speed. Multiple instances of the same image were captured while bracketing exposure −1 and −2 steps under default settings to ensure suitably exposed images were acquired in both UV and VIS. As the Nikkor lens does not allow for autofocusing functionality, a 25–55 mm focusing helicoid was used to allow manual focusing via the live‐view feature. No focus shift was observed between lenses/image types, and so the image was focused originally using the visible spectrum, photographed and then the UV/IR cut filter was removed and the UV filter attached without loss of focus.

#### 2.2.3 Staging

**FIGURE 2:**
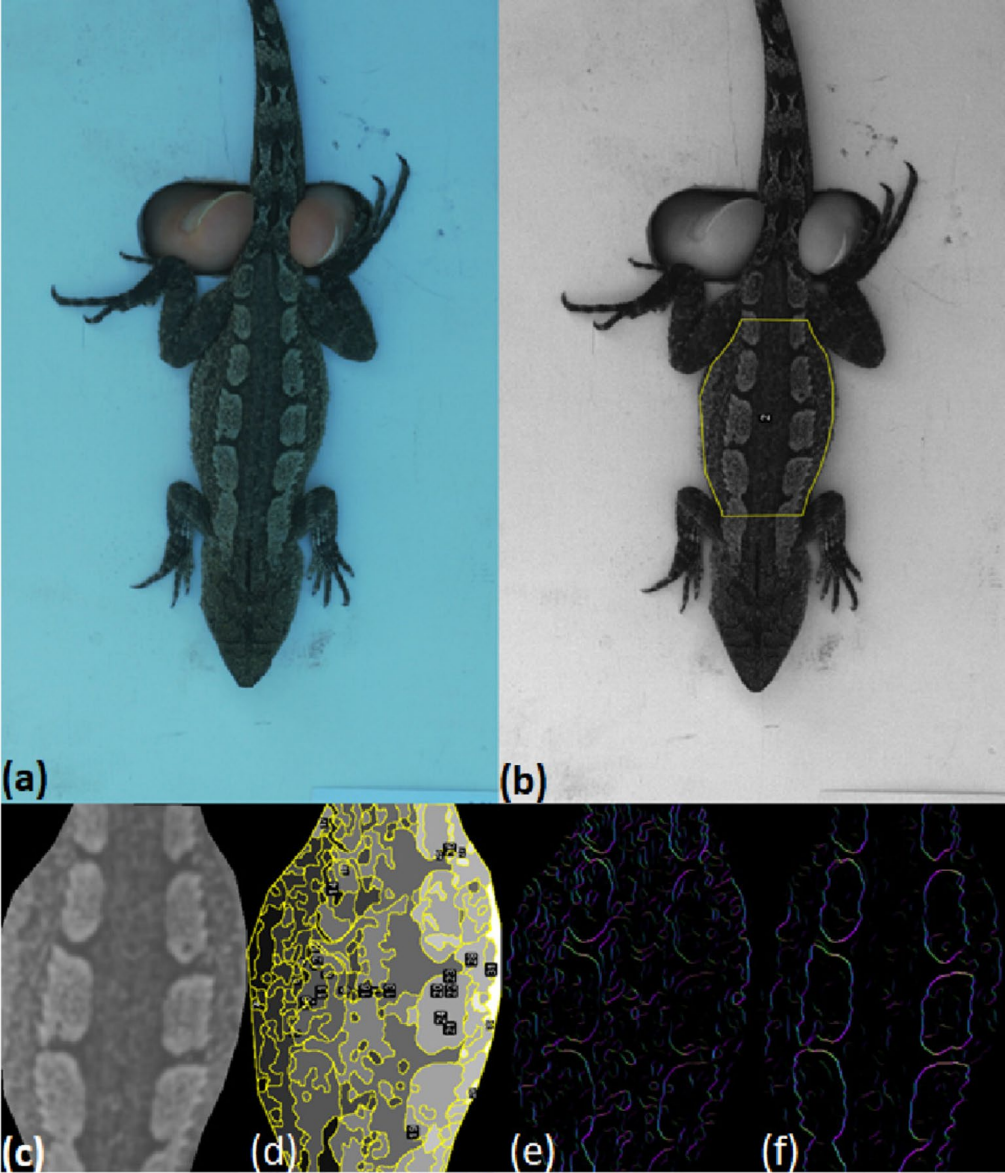
Example of image processing stages using the micaToolbox suite for ImageJ software. (a) Jacky dragon placed on custom‐build stage for photography of dorsal patterns. (b) Multispectral image created combining VIS and UV photographs and example ROI between fore‐ and hind limbs. (c) Estimated information available based on observer visual traits (spectral sensitivities, acuity) and viewing distance; in this case, a grey butcherbird model at 1 m. (d–f) Visualisations of: (d) detectable clusters, (e) colour edge intensity and (f) luminance edge intensity.

The camera was mounted to a 6‐ft tripod (Manfrotto 190XB), fully extended for photography. The camera pointed directly down, looking onto a stage that sat 200 mm off the ground where the lizard could be held for photography (Figure 2a). The front of the lens was positioned approximately 1500 mm perpendicular to the lizard, which fills an adequate region of the frame, as the Nikkor EL 80 mm f/5.6 is an enlarger lens (×5). Individual lizards were always photographed in full shade, or when natural shade was not available, an 800 mm diameter photography reflector with the reflective side outwards was used to provide diffuse shade in the scene. The camera was triggered using a remote trigger, and after capturing VIS spectrum images, the filter was replaced and UV spectrum images of the exact scene were taken. If the lizard moved mid‐process, the series was abandoned and restarted. A scale was also included in each image.

### 2.3 Image Analysis

We used the micaToolbox framework (v2.2, Troscianko and Stevens 2015) for the ImageJ software suite (v1.53e, Schneider et al. 2012) for image processing, visual system modelling and simulation and analysis. This software offers a range of options for the analysis of pattern features, observer visual systems and conspicuousness‐relevant metrics. For each lizard, the VIS and UV images (RAW format) of the same scene were combined into a multi‐spectrum image (16‐bit). The dorsal area of the lizard in the scene was defined using the polygon tool in ImageJ and denoted as the area of its dorsal surface between the front and hind legs (Figure 2b), with the scale of each lizard determined using the ruler visible in each image.

The dorsal ROI in each image was then analysed multiple times from the perspective of different observers and viewing distances. The visual capabilities of three potential observers of the lizards were used and are defined by cone types and their relative absorption profiles, as well as visual acuity values (cycles per degree, cpd). We modelled the visual capabilities of Jacky dragons ( *A. muricatus* ), grey butcherbirds ( *C. torquatus* ) and laughing kookaburras (*Dacelo novaeguineae*) (Figure S1). The spectral sensitivities of Jacky dragons have not been determined, but as colour vision is likely to be similar for agamid lizards, we used the profiles for *C. decresii* (Yewers et al. 2015) featuring 5 cone types (see Figure S1 for details) and set acuity to 7.4 cpd, which has been determined for Jacky dragons (New 2012). We wished to model potential avian predators and selected species that are known to prey upon Jacky dragons (laughing kookaburra, Allen et al. 2009) or are predators of lizards and co‐occur with Jacky dragons (grey butcherbird, McLean et al. 2014). These two predator models were investigated as they possess violet‐sensitive (VS) and ultraviolet‐sensitive (UVS) visual systems, respectively. In the absence of specific cone sensitivities for these birds, we used generalised models for VS and UVS avian species (Endler and Mielke 2005) and set acuity at 41 cpd for kookaburras (Moroney and Pettigrew 1987) and 30 cpd for butcherbirds (as an approximation in the absence of data for this species).

The multispectral images were converted to cone catch models using a combination of the spectral sensitivities of the chosen observer, illuminant data (300–700 nm forest daylight), grey standard and camera calibration data. The ability to resolve patterns is constrained by visual acuity and will vary as a function of viewing distance. Consequently, for each observer we repeated the analysis of each lizard pattern and varied the simulated viewing distance by using built‐in QCPA tools (van den Berg et al. 2020) for modelling spatial acuity at 1 and 5 m. The size of the dorsal pattern ROI at a viewing distance of 5 m when observed by a lizard observer was insufficient to provide useful results, so this set of manipulations was not undertaken. Each lizard dorsal pattern was therefore analysed using QCPA visual modelling 5 times (twice each for avian predator observers and once for lizard observers). Gaussian acuity correction (non‐square ROIs), negative replacement values of 0.001 and Weber fraction estimates of 0.05 (in the absence of specific data, Vorobyev et al. 2001) were used. By implementing the QCPA framework, we obtained multiple measures for each observer and viewing distance combination: cluster statistics, local edge intensity and multiple colour pattern measurements, including colour adjacency analysis (CAA), boundary strength analysis (BSA) and visual contrast analysis (VCA). A full description of the output generated by QCPA is available elsewhere (see van den Berg et al. 2020). Briefly, the ROI is degraded by several passes of Weber filters and JND filters (just‐noticeable differences) based on acuity and cone information to simulate the information available to the modelled observer (Figure 2c–f). The process then measures pattern and conspicuousness‐relevant metrics such as discernible cluster count, edge intensity (both colour and luminance) and numerous complexity measures, among others (Table A1), specifying a JND of 2 during the analytical process.

### 2.4 Statistical Analysis

Data were examined in different ways as outlined below, with all analyses undertaken in the R Statistical Environment (R Core Team 2020). We focussed our analysis on whether pattern information varied within observers separately for viewing distances of 1 and 5 m. We explored graphically significant factors or interactions in each model by generating estimated marginal means and 95% confidence intervals. Direct comparisons between observers were also considered and are described in the Appendix S1, with outcomes reported in Tables S1–S3 and significant effects depicted in Figures S2 and S3.

#### 2.4.1 Variation Within Observers at 1 m

We examined variation in dorsal patterns at a viewing distance of 1 m separately for lizard, butcherbird and kookaburra observers as a function of sex (male, female), habitat (coastal heath, other), clade (A, B, C, D) and SVL. The number of clusters and counts within clusters were examined using a generalised linear model (*glm*) with a Poisson distribution and a log link function. Models examining the mean colour intensity and luminance intensity were fitted with Gaussian distributions but required square‐root transformation of the response variables. As we had no a priori expectation about which of the colour pattern variables generated by the QCPA framework are worthy of close attention, we undertook principal components analysis to reduce the dataset. For each observer, we extracted the colour pattern variables and used *prcomp* to identify the principal components (variables were centred and scaled before analysis). We then used the function *PCAtest* from the library of the same name (Camargo 2021) to identify which components had statistical support and examined the first three components (PC1‐3) for each observer (as there was justification to do so). Models were fit with Gaussian distributions, and transformations were not required for PC1–3 for any of the observers. For all response variables, we fit models with interaction terms sex × habitat and sex × clade as well as SVL. The significance of models was first assessed with the *anova* function, applying Type I sums of squares calculations to test interaction terms before the *Anova* function from the *car* package (Fox and Weisberg 2018) was used to test main effects, implementing Type III sums of squares if at least one interaction term was significant (non‐significant interaction terms removed) or Type II sums of squares when there were no significant interactions (interaction terms removed).

#### 2.4.2 Variation Within Observers at 5 m

We examined variation in dorsal patterns at a viewing distance of 5 m but focused only on the avian predators, as the ability to resolve spatial detail is greatly reduced for lizards at this viewing distance due to their much lower spatial acuity level. Our general approach was the same as for investigations at 1 m viewing distance outlined above and examined clusters and counts within clusters, colour and luminance intensity and reducing colour pattern variables using principal components analysis. Model construction and testing the significance of models was also the same as that undertaken for 1 m viewing distance.

## 3 Results

Throughout the results we report pattern information available to individual observers. These data are generated by the framework we have used to quantify dorsal patterns of Jacky dragons and are predictions based on assumptions of visual capabilities of the nominated observers as described in the methods section. Our terminology below simplifies this to suggest what observers can see, but these should be read as shorthand for predictions of what visual information is potentially available to the animals.

### 3.1 Variation Within Observer at 1 m

**FIGURE 3:**
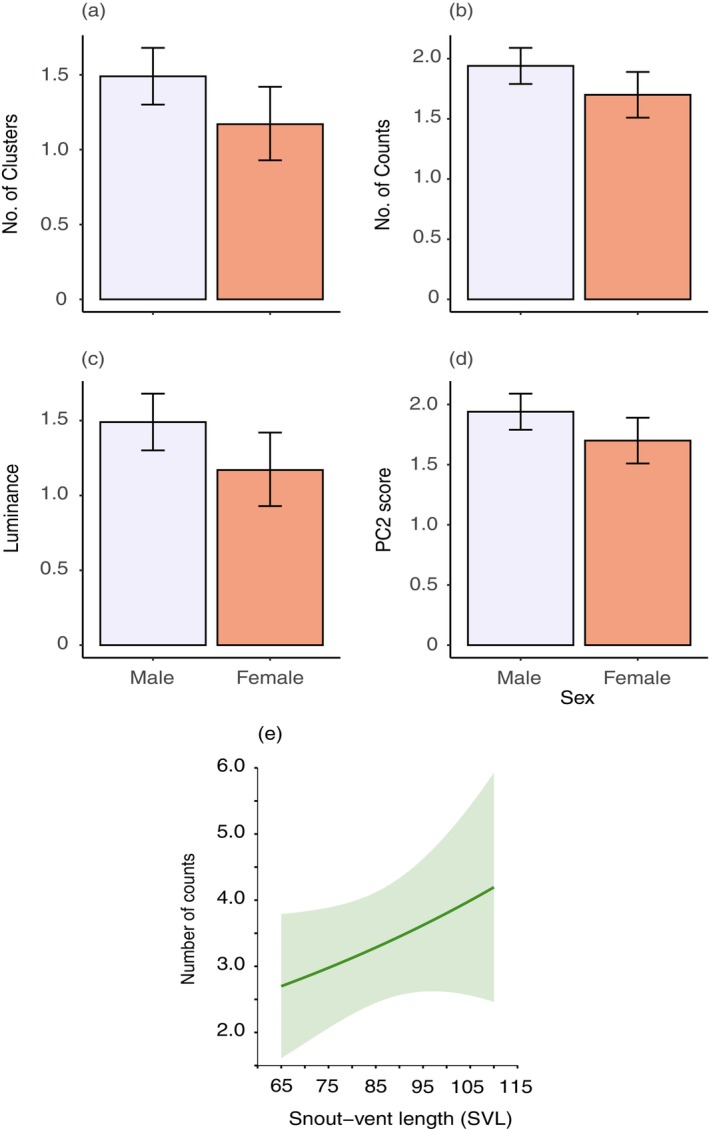
Estimated marginal means predicted from regression models for lizard observers at 1 m viewing distance. Sex differences were found for the (a) number of clusters and (b) counts within clusters, as well as for (c) luminance and (d) PC2 score. Error bars in (a)–(d) are 95% Cis, with males shown in lavender and females in salmon colours. (e) The number of counts within clusters was also found to vary as a function of snout‐vent length (SVL). The predicted value at each SVL is given by the solid line, while the shaded region represents the standard error of the predicted values.

**FIGURE 4:**
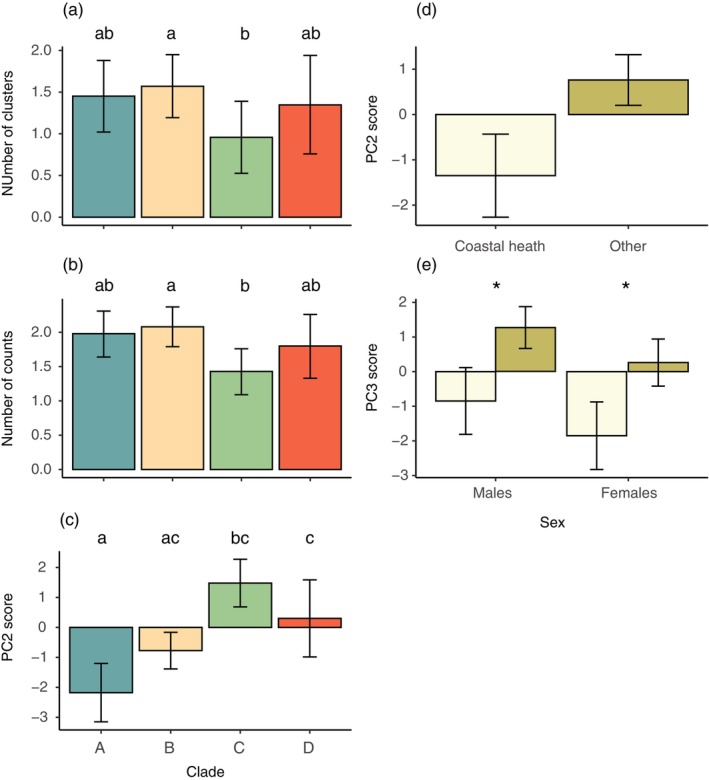
Estimated marginal means predicted from regression models for lizard observers at 1 m viewing distance. An effect of clade was found for the (a) number of clusters and (b) counts within clusters, as well as for (c) PC2 score. PC2 score also varied as a function of habitat (d), while PC3 score varied as a function of the interaction between habitat and sex (e), with coastal heath shown in light green and the other in dark green. Error bars are 95% CIs. Different letters above bars in (a–c), or * in (e), indicated pairwise differences.

All modelled animal observers showed variation in visible pattern features at a viewing distance of 1 m. Principal component analysis was used to reduce colour pattern variables (summarised in Table B1), with three components supported for each observer. For lizard observers, PC1 reflected overall diversity and complexity, with high scores indicating low pattern diversity and complexity. PC2 and PC3 were more specific, with high PC2 scores indicative of low luminance contrast but high colour and RNL contrast, and high PC3 scores representing low boundary strength values. The results of statistical modelling for lizard observers are summarised in Table 1, with all predictor variables proving to significantly influence outcomes for one or more variables. Lizard observers yielded significant effects of sex (cluster, counts, mean luminance intensity, PC2), clade (clusters, counts, PC2), habitat type (PC2) and SVL (counts), while also exhibiting a sex × habitat type interaction effect for PC3. Compared with females, male lizards exhibited significantly more clusters and counts (Figure 3a,b respectively), higher luminance contrast (Figure 3c) and higher PC2 scores (Figure 3d). Larger lizards exhibited a higher number of counts (Figure 3e), while the clade to which lizard patterns belong was restricted to pairwise differences between clades B and C for cluster and count data (Figure 4a,b; Table C1). However, more variability between clades was evident for PC2 scores (Figure 4c). Lizards from coastal heath habitats exhibited significantly lower PC2 scores (Figure 4d), while the effect of habitat on PC3 scores was part of a significant interaction with sex. On closer inspection, the direction of the effect was the same, with coastal heath lizards scoring significantly lower than lizards from other habitats (Figure 4e).

**TABLE 1:** Statistical outcomes for analysis of lizard dorsal patterns using visual traits modelling Jacky dragon ( *Amphibolurus muricatus* ) observer at 1 m.

Terms	SS type	df	Test statistic^a^	Residual df	Residual dev.	*p*
Clusters						
**Sex**	**II**	**1**	**5.035**			**0.025**
Habitat	II	1	0.410			0.522
**Clade**	**II**	**3**	**8.692**			**0.034**
SVL	II	1	1.818			0.178
Sex × habitat	I	1	0.088	52	49.646	0.767
Sex × clade	I	3	3.804	49	45.843	0.283
Counts						
**Sex**	**II**	**1**	**4.7022**			**0.030**
Habitat	II	1	0.7042			0.401
**Clade**	**II**	**3**	**16.6651**			**0.001**
**SVL**	**II**	**1**	**4.8912**			**0.027**
Sex × habitat	I	1	0.1757	52	93.96	0.675
Sex × clade	I	3	4.9207	49	89.04	0.177
Edge intensity—colour						
Sex	II	1	3.736			0.053
Habitat	II	1	0.029			0.866
Clade	II	3	4.894			0.180
SVL	II	1	1.074			0.300
Sex × habitat	I	1	0.007	52	0.252	0.226
Sex × clade	I	3	0.009	49	0.243	0.614
Edge intensity—luminance						
**Sex**	**II**	**1**	**5.506**			**0.019**
Habitat	II	1	0.0963			0.756
Clade	II	3	2.457			0.483
SVL	II	1	0.0007			0.978
Sex × habitat	I	1	0.0055	52	0.29222	0.310
Sex × clade	I	3	0.0283	49	0.26383	0.153
Principal component 1 (PC1)						
Sex	II	1	2.797			0.094
Habitat	II	1	0.521			0.470
Clade	II	3	5.760			0.124
SVL	II	1	1.200			0.273
Sex × habitat	I	1	8.554	52	243.010	0.164
Sex × clade	I	3	26.107	49	216.900	0.117
Principal component 2 (PC2)						
**Sex**	**II**	**1**	**10.046**			**0.002**
**Habitat**	**II**	**1**	**11.573**			**0.001**
**Clade**	**II**	**3**	**32.757**			**0.001**
SVL	II	1	0.405			0.525
Sex × habitat	I	1	3.004	52	72.136	0.137
Sex × clade	I	3	5.519	49	66.617	0.255
Principal component 3 (PC3)						
Sex	III	1	2.5858			0.108
**Habitat**	**III**	**1**	**11.4015**			**0.001**
Clade	III	3	5.8981			0.117
SVL	III	1	0.9981			0.318
**Sex × habitat**	**I**	**1**	**8.234**	**52**	**77.326**	**0.020**
Sex × clade	I	3	2.72	49	74.606	0.618

*Note:* Bold denotes significant outcome.

^a^ Deviance for SS type I and chi‐square for SS type II or III.

**FIGURE 5:**
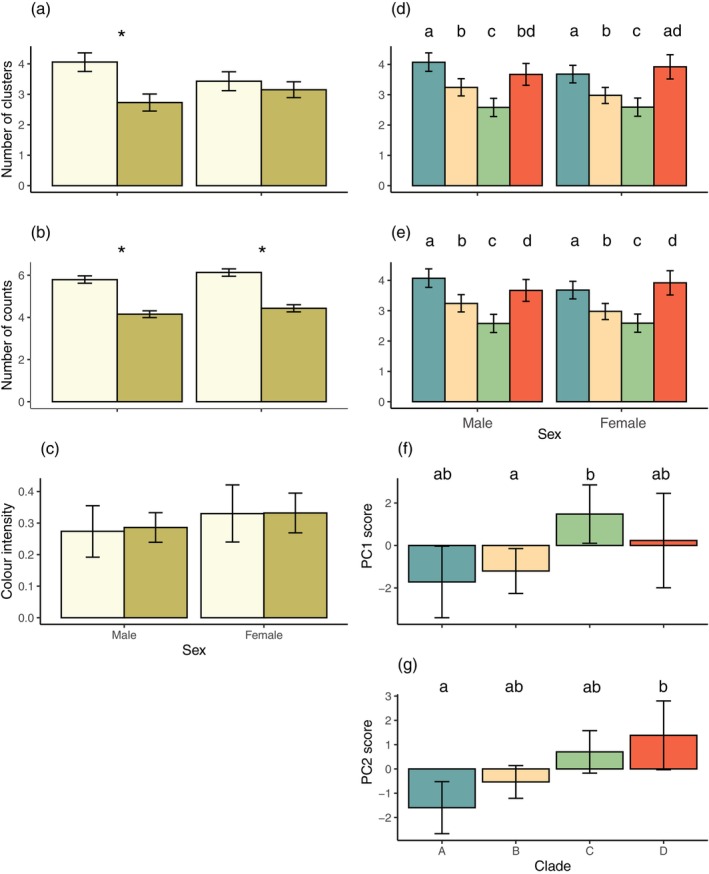
Estimated marginal means predicted from regression models for butcherbird observers at 1 m viewing distance. A significant interaction between habitat and sex was found for the (a) number of clusters and (b) counts within clusters, as well as for (c) colour intensity. A significant interaction between clade and sex was found for (d) the number of clusters and (e) counts within clusters, while an effect of clade was present for (f) PC1 and (g) PC2 scores. In (a)–(c) coastal heath is shown in light green and other habitats in dark green colours. Error bars are 95% CIs. An * in (a–c) and different letters above bars in (d–g) indicate pairwise differences (contrasts within sex for d, e).

**FIGURE 6:**
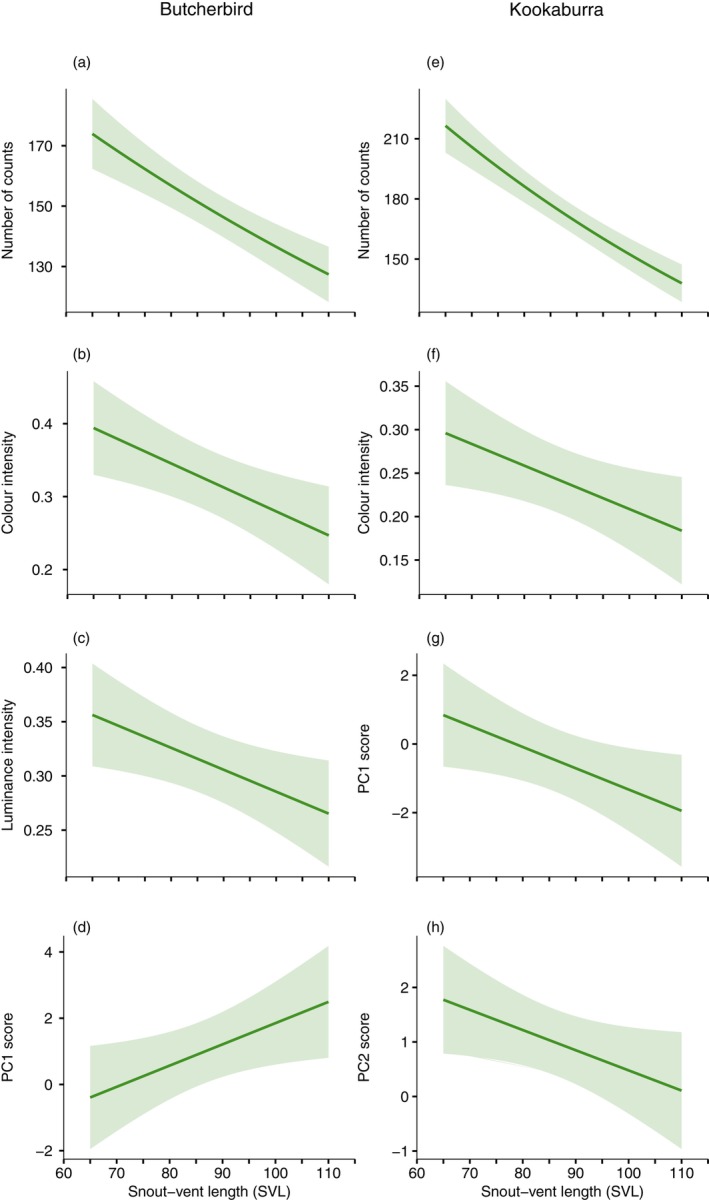
Predicted relationship between snout‐vent length (SVL) and measured variables for butcherbird (left column) and kookaburra (right column) observers. For butcherbirds, a significant decrease was found for (a) the number of counts within clusters, (b) colour and (c) luminance intensity, while (d) PC1 score was found to increase with SVL. For kookaburras, a significant negative relationship was found for (e) the number of counts within clusters, (f) colour intensity, (g) PC1 and (h) PC2 scores. The predicted value at each SVL is given by the solid line, while the shaded region represents the standard error of the predicted values.

For butcherbirds, high PC1 scores indicate low pattern diversity and complexity, high PC2 scores indicate high colour and RNL contrast, and high PC3 scores represent low boundary strength values (Table B1). When modelling the grey butcherbird at 1 m, we again found all variables except PC3 to be significantly affected by one or more predictors (Table 2). We found effects of sex (clusters, counts), clade (clusters, counts, PC1, PC2), habitat type (clusters, counts) and SVL (counts, mean colour intensity, mean luminance, PC1), as well as interaction effects for sex × habitat type (clusters, counts, mean colour intensity) and sex × clade (clusters, counts). We examined the effect of habitat within sex and found that butcherbirds were able to perceive more clusters and counts within those clusters for coastal heath lizards compared with lizards from other habitat types (Figure 5a,b). Both measures yielded significant contrasts for males, while pairwise contrasts were only significant for female counts within clusters (see Table C2). There was no difference in colour intensity between lizards from different habitat types for either male or female lizards (Figure 5c). We also examined differences between clades separately for males and females, with all pairwise contrasts significant except for cluster comparisons between clades B and D for males and A and D for females (Figure 5d,e; Table C3). Significant pairwise contrasts for the main effect of clade for PC1 were restricted to clades B and C for PC1 and between clades A and D for PC2 (Figure 5f,g). Finally, larger lizards were associated with fewer counts, lower mean colour and luminance intensity and higher PC1 values (Figure 6a–d).

**TABLE 2:** Statistical outcomes for analysis of lizard dorsal patterns using visual traits modelling grey butcherbirds ( *Cracticus torquatus* ) observer at 1 m.

Terms	SS type	df	Test statistic^a^	Residual df	Residual dev.	*p*
Clusters						
**Sex**	**III**	**1**	**5.081**			**0.024**
**Habitat**	**III**	**1**	**21.045**			**< 0.001**
**Clade**	**III**	**3**	**61.589**			**< 0.001**
SVL	III	1	0.815			0.367
**Sex × habitat**	**I**	**1**	**59.84**	**52**	**372.90**	**< 0.001**
**Sex × clade**	**I**	**3**	**11.729**	**49**	**361.17**	**0.008**
Counts						
**Sex**	**III**	**1**	**4.356**			**0.037**
**Habitat**	**III**	**1**	**96.480**			**< 0.001**
**Clade**	**III**	**3**	**145.809**			**< 0.001**
**SVL**	**III**	**1**	**33.186**			**< 0.001**
**Sex × habitat**	**I**	**1**	**372.84**	**52**	**2476.5**	**< 0.001**
**Sex × clade**	**I**	**3**	**101.71**	**49**	**2374.8**	**< 0.001**
Edge intensity—Colour						
Sex	III	1	1.774			0.183
Habitat	III	1	0.952			0.329
Clade	III	3	2.251			0.522
**SVL**	**III**	**1**	**9.118**			**0.003**
**Sex × habitat**	**I**	**1**	**0.040**	**52**	**0.316**	**0.010**
Sex × clade	I	3	0.019	49	0.297	0.378
Edge intensity—luminance						
Sex	II	1	0.026			0.871
Habitat	II	1	0.207			0.649
Clade	II	3	4.978			0.173
**SVL**	**II**	**1**	**5.026**			**0.025**
Sex × habitat	I	1	0.006	52	0.118	0.180
Sex × clade	I	3	0.002	49	0.176	0.919
Principal component 1 (PC1)						
Sex	II	1	0.003			0.955
Habitat	II	1	0.264			0.607
**Clade**	**II**	**3**	**11.045**			**0.011**
**SVL**	**II**	**1**	**4.648**			**0.031**
Sex × habitat	I	1	7.892	52	216.77	0.176
Sex × clade	I	3	5.597	49	211.17	0.729
Principal component 2 (PC2)						
Sex	II	1	2.146			0.143
Habitat	II	1	0.128			0.910
**Clade**	**II**	**3**	**21.643**			**< 0.001**
SVL	II	1	1.914			0.167
Sex × habitat	I	1	4.243	52	86.885	0.112
Sex × clade	I	3	4.484	49	82.401	0.446
Principal component 3 (PC3)						
Sex	II	1	1.031			0.310
Habitat	II	1	0.124			0.724
Clade	II	3	3.062			0.382
SVL	II	1	0.821			0.365
Sex × habitat	I	1	1.801	52	87.031	0.300
Sex × clade	I	3	5.016	49	82.016	0.392

*Note:* Bold denotes significant outcome.

^a^ Deviance for SS type I and chi‐square for SS type II or III.

**FIGURE 7:**
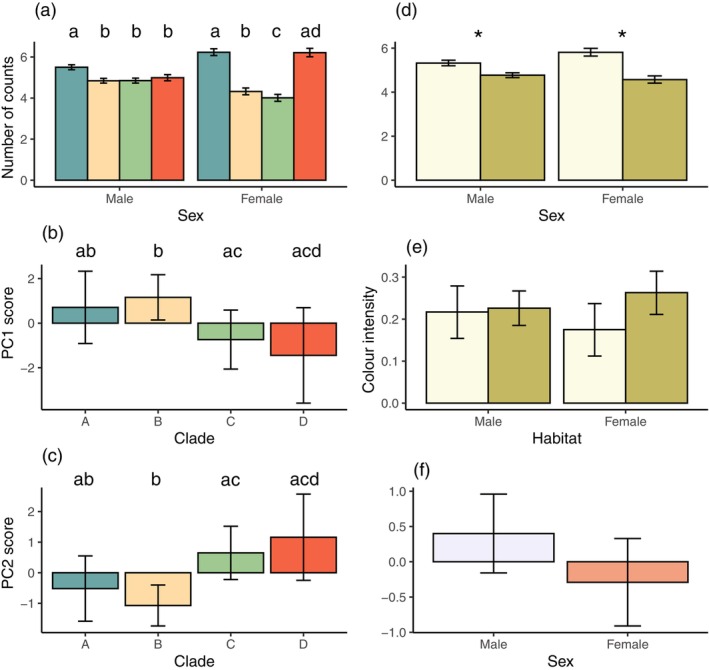
Estimated marginal means predicted from regression models for kookaburra observers at 1 m viewing distance. A significant interaction between clade and sex was found for the (a) number of counts within clusters, while (b) PC1 and (c) PC2 scores varied as a function of clade. A significant interaction between sex and habitat was found for (d) counts within clusters and (e) colour intensity, while a (f) sex effect was found for PC2 score. In (a) and (d)–(f) males are shown in lavender and females in salmon colours. Error bars are 95% CIs. Different letters above bars in (a–c), and an * in (d, e) indicate pairwise differences (contrasts within sex for d and e).

Principal component analysis for laughing kookaburras differed from that for butcherbirds (Table B1). High PC1 scores indicate higher pattern diversity and complexity. PC2 presents lower colour and RNL contrast with higher values, while higher PC3 results suggest lower boundary strength. Overall kookaburra perception of lizard patterns revealed all variables except clusters and PC3 being significantly affected by at least one factor (Table 3; see Table C4 for pairwise contrast outcomes). This resulted in effects of sex (counts, PC2), clade (counts, PC1, PC2), habitat type (counts) and SVL (counts, mean colour intensity, PC1, PC2), and interaction effects for sex × habitat type (counts, mean colour intensity) and sex × clade (counts). Multiple pairwise differences between clades within sex for count data (Figure 7a) were identified, while contrasts between clades for PC1 and PC2 were limited to B‐C and B‐D (Figure 7b,c). Examining the effect of habitat within sex (see Table C5) showed coastal heath lizards were perceived to feature higher counts for both males and females (Figure 7d), though no differences were found for mean colour intensity (Figure 7e). The main effect of sex for PC2 reflects significantly lower PC2 scores for females (Figure 7f), which indicates greater colour and RNL contrast. Finally, larger lizards were observed by kookaburras to have lower counts, colour intensity and values for PC1 and PC2 (Figure 6e–h, respectively).

**TABLE 3:** Statistical outcomes for analysis of lizard dorsal patterns using visual traits modelling Laughing kookaburra (*Dacelo novaeguineae*) observer at 1 m.

Terms	SS type	df	Test statistic^a^	Residual df	Residual dev.	*p*
Clusters						
Sex	II	1	0.095			0.757
Habitat	II	1	0.007			0.935
Clade	II	3	1.490			0.685
SVL	II	1	0.141			0.708
Sex × habitat	I	1	0.062	52	22.399	0.803
Sex × clade	I	3	0.014	49	22.385	0.999
Counts						
**Sex**	**III**	**1**	**27.887**			**< 0.001**
**Habitat**	**III**	**1**	**23.456**			**< 0.001**
**Clade**	**III**	**3**	**112.028**			**< 0.001**
**SVL**	**III**	**1**	**78.079**			**< 0.001**
**Sex × habitat**	**III**	**1**	**11.463**			**< 0.001**
**Sex × clade**	**III**	**3**	**97.674**			**< 0.001**
Edge intensity—Colour						
Sex	**III**	1	2.663			0.103
Habitat	**III**	1	0.050			0.823
Clade	**III**	3	1.654			0.647
**SVL**	**III**	**1**	**5.436**			**0.020**
**Sex × habitat**	**III**	**1**	**3.917**			**0.047**
Sex × clade	I	3	0.017	49	0.265	0.362
Edge intensity—luminance						
Sex	II	1	0.040			0.842
Habitat	II	1	0.083			0.774
Clade	II	3	6.448			0.092
SVL	II	1	3.977			0.051
Sex × habitat	II	1	3.028			0.088
Sex × clade	I	3	0.001	49	0.190	0.971
Principal component 1 (PC1)						
Sex	II	1	1.408			0.235
Habitat	II	1	0.249			0.618
**Clade**	**II**	**3**	**10.562**			**0.0145**
**SVL**	**II**	**1**	**4.664**			**0.031**
Sex × habitat	I	1	5.530	52	203.04	0.242
Sex × clade	I	3	4.743	49	198.30	0.760
Principal component 2 (PC2)						
**Sex**	**II**	**1**	**3.972**			**0.046**
Habitat	II	1	0.152			0.697
**Clade**	**II**	**3**	**18.578**			**< 0.001**
**SVL**	**II**	**1**	**3.847**			**0.0498**
Sex × habitat	I	1	1.244	52	89.110	0.397
Sex × clade	I	3	4.314	49	84.796	0.477
Principal component 3 (PC3)						
Sex	II	1	0.011			0.916
Habitat	II	1	2.071			0.150
Clade	II	3	4.094			0.252
SVL	II	1	1.429			0.232
Sex × habitat	I	1	5.084	52	100.049	0.105
Sex × clade	I	3	5.304	49	94.741	0.433

*Note:* Bold denotes significant outcome.

^a^ Deviance for SS type I and chi‐square for SS type II or III.

### 3.2 Variation Within Observers at 5 m

**FIGURE 8:**
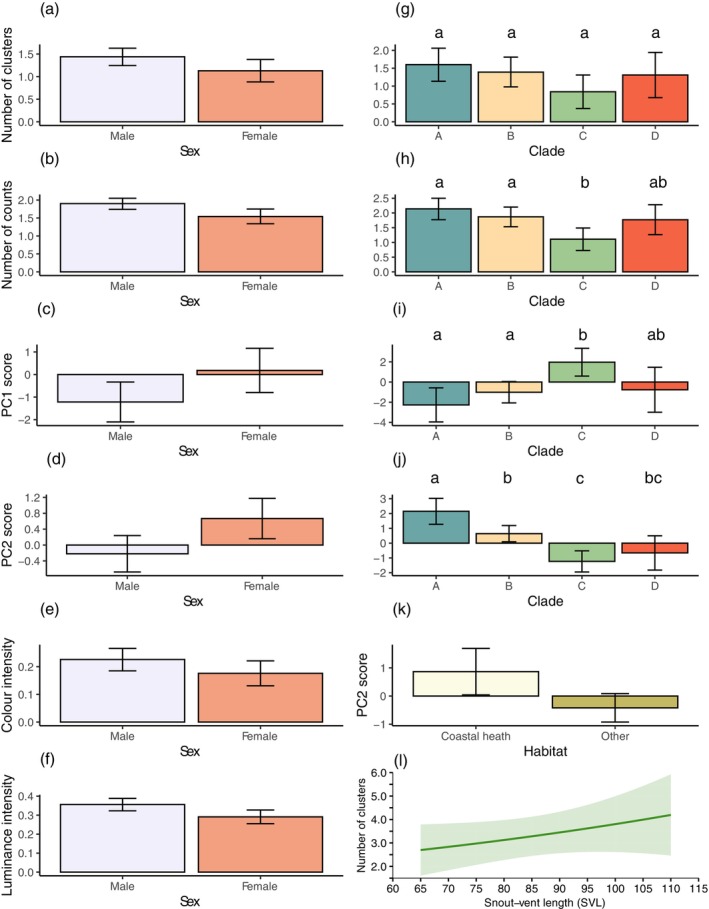
Estimated marginal means predicted from regression models for butcherbird observers at 5 m viewing distance. A significant effect of sex was found for (a) number of clusters, (b) number of counts within clusters, (c) PC1 and (d) PC2 scores, as well as (e) colour and (f) luminance intensity. A significant effect of clade was found for (g) number of clusters, (h) number of counts within clusters, (i) PC1 and (j) PC2 scores, while (k) PC2 score varied by habitat. Error bars in (a)–(k) are 95% CIs. (l) The number of clusters varied as a function of snout‐vent length (SVL). The predicted value at each SVL is given by the solid line, while the shaded region represents the standard error of the predicted values. Different letters above bars in (g–j) indicate pairwise differences.

Variation in pattern detail was still apparent for avian observers at a viewing distance of 5 m. Principal component analysis reduced colour and pattern variables (Table B1), with support for two principal components for both avian observers. For butcherbirds, high PC1 values suggest lower pattern diversity and complexity, while high PC2 scores indicate higher pattern contrast. Here we found effects on pattern features for sex (clusters, counts, mean luminance, mean colour intensity, PC1, PC2), clade (clusters, counts, PC1, PC2), habitat type (PC2) and SVL (clusters) (Table 4). Butcherbirds saw more clusters, higher counts within clusters, higher luminance, higher colour intensity, but lower PC1 and PC2 values in males compared to females (Figure 8a–f). Pairwise contrasts for clade main effects (see Table D1) indicated contrasts between clades A and C and between B and C to be significant for counts and PC1 (Figure 8h,i), while all contrasts except B and D, as well as C and D, were significantly different for PC2 (Figure 8j). Coastal heath lizards had significantly higher PC2 values than lizards from other habitat types (Figure 8k), while larger lizards displayed more clusters than smaller lizards (Figure 8l).

**TABLE 4:** Statistical outcomes for analysis of lizard dorsal patterns using visual traits modelling grey butcherbirds (*Cracticus torquatus)* observer at 5 m.

Terms	SS type	df	Test statistic^a^	Residual df	Residual dev.	*p*
Clusters						
**Sex**	**II**	**1**	**4.763**			**0.029**
Habitat	II	1	1.142			0.285
**Clade**	**II**	**3**	**8.183**			**0.042**
**SVL**	**II**	**1**	**5.041**			**0.025**
Sex × habitat	I	1	0.068	52	50.796	0.794
Sex × clade	I	3	3.449	49	47.347	0.327
Counts						
**Sex**	**II**	**1**	**9.418**			**0.002**
Habitat	II	1	1.595			0.207
**Clade**	**II**	**3**	**22.052**			**< 0.001**
SVL	II	1	1.842			0.175
Sex × habitat	I	1	0.460	52	131.45	0.498
Sex × clade	I	3	3.719	49	127.73	0.294
Edge intensity—colour						
**Sex**	**II**	**1**	**3.864**			**0.049**
Habitat	II	1	0.041			0.841
Clade	II	3	6.103			0.107
SVL	II	1	0.182			0.670
Sex × habitat	I	1	0.0005	52	0.474	0.824
Sex × clade	I	3	0.008	49	0.465	0.829
Edge intensity—luminance						
**Sex**	**II**	**1**	**10.289**			**0.001**
Habitat	II	1	0.098			0.754
Clade	II	3	3.492			0.322
SVL	II	1	0.306			0.580
Sex × habitat	I	1	0.003	52	0.308	0.471
Sex × clade	I	3	0.028	49	0.276	0.173
Principal component 1 (PC1)						
**Sex**	**II**	**1**	**6.508**			**0.011**
Habitat	II	1	1.924			0.165
**Clade**	**II**	**3**	**12.121**			**0.007**
SVL	II	1	0.737			0.391
Sex × habitat	I	1	2.073	52	223.98	0.493
Sex × clade	I	3	7.379	49	216.60	0.644
Principal component 2 (PC2)						
**Sex**	**II**	**1**	**9.716**			**0.002**
**Habitat**	**II**	**1**	**5.278**			**0.022**
**Clade**	**II**	**3**	**39.343**			**< 0.001**
SVL	II	1	1.738			0.187
Sex × habitat	I	1	0.407	52	60.479	0.558
Sex × clade	I	3	2.345	49	58.133	0.577

*Note:* Bold denotes significant outcome.

^a^ Deviance for SS type I and chi‐square for SS type II or III.

**FIGURE 9:**
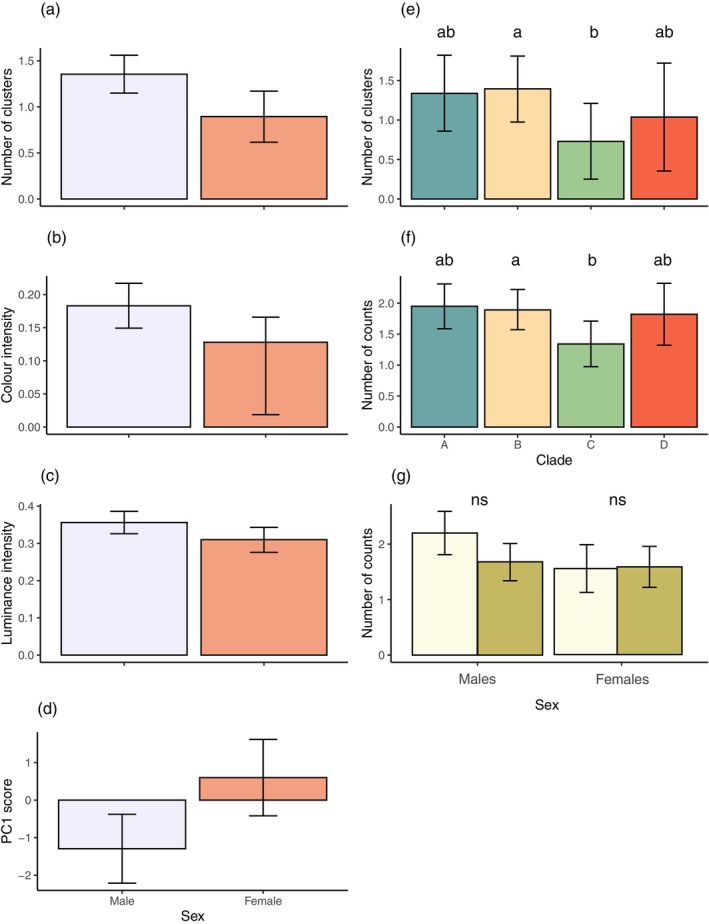
Estimated marginal means predicted from regression models for kookaburra observers at 5 m viewing distance. A significant effect of sex was found for (a) number of clusters, (b) colour and (c) luminance intensity as well as (d) PC1 scores. (e) The number of clusters and (f) counts within clusters varied by clade, while the (g) number of counts within clusters varied by sex and habitat, with coastal heath shown in light green and others in dark green colours. Different letters above bars in (e–f) indicate pairwise differences.

For laughing kookaburras, high PC1 scores were indicative of low diversity and complexity in bodily colouration, while higher PC2 outcomes suggest high contrast but lower boundary strength (Table B1). We identified main effects of sex (clusters, counts, mean luminance, mean colour intensity, PC1) and clade (clusters, counts) and an interaction effect of sex × habitat type for counts (Table 5). Males exhibited significantly more clusters, higher colour and luminance intensity values and lower PC1 scores (Figure 9a–d, respectively). For both clusters and counts, only pairwise comparisons between clades B‐C were significant (Table D2), with B presenting higher values in both measures (Figure 9e,f). We examined the effect of habitat on counts separately for each sex, but although coastal heath lizards had higher counts for each sex (Figure 9g), pairwise contrasts were not significant within each sex (Table D2).

**TABLE 5:** Statistical outcomes for analysis of lizard dorsal patterns using visual traits modelling laughing kookaburra (*Dacelo novaeguineae*) observer at 5 m.

Terms	SS type	df	Test statistic^a^	Residual df	Residual dev.	*p*
Clusters						
**Sex**	**II**	**1**	**8.823**			**0.003**
Habitat	II	1	0.826			0.363
**Clade**	**II**	**3**	**9.958**			**0.019**
SVL	II	1	0.433			0.511
Sex × habitat	I	1	1.970	52	42.258	0.160
Sex × clade	I	3	2.225	49	40.034	0.527
Counts						
**Sex**	**III**	**1**	**16.318**			**< 0.001**
Habitat	III	1	2.393			0.122
**Clade**	**III**	**3**	**10.294**			**0.016**
SVL	III	1	0.708			0.400
**Sex × habitat**	**I**	**1**	**5.696**	**52**	**102.918**	**0.017**
Sex × clade	I	3	5.011	49	97.906	0.171
Edge intensity—colour						
**Sex**	**II**	**1**	**6.946**			**0.008**
Habitat	II	1	0.043			0.836
Clade	II	3	4.450			0.217
SVL	II	1	1.244			0.265
Sex × habitat	I	1	0.004	52	0.327	0.424
Sex × clade	I	3	0.009	49	0.318	0.717
Edge intensity—Luminance						
**Sex**	**II**	**1**	**6.185**			**0.013**
Habitat	II	1	0.226			0.635
Clade	II	3	3.581			0.310
SVL	II	1	0.416			0.519
Sex × habitat	I	1	0.004	52	0.257	0.329
Sex × clade	I	3	0.026	49	0.230	0.132
Principal component 1 (PC1)						
**Sex**	**II**	**1**	**11.059**			**< 0.001**
Habitat	II	1	0.436			0.509
Clade	II	3	7.802			0.050
SVL	II	1	0.057			0.812
Sex × habitat	I	1	0.153	52	243.06	0.857
Sex × clade	I	3	14.079	49	228.98	0.390
Principal component 2 (PC2)						
Sex	II	1	1.152			0.283
Habitat	II	1	1.043			0.307
Clade	II	3	5.697			0.127
SVL	II	1	3.405			0.065
Sex × habitat	I	1	1.305	52	113.10	0.448
Sex × clade	I	3	2.201	49	110.90	0.808

*Note:* Bold denotes significant outcome.

^a^ Deviance for SS type I, chi‐square for SS type II or III.

## 4 Discussion

This study quantified the dorsal patterns of Jacky dragon ( *A. muricatus* ) lizards across their geographic range and from the perspective of conspecifics and potential predators. Our results support the hypotheses that their dorsal patterns vary due to size, sex, habitat and phylogenetic relatedness. In addition, our approach revealed the importance of taking receiver sensory capabilities into account, as variation in pattern detail depended on the observer viewing the lizard and the distance at which animal patterns are viewed, with information available to lizard and avian predators differing in ecologically relevant and interesting ways.

Several aspects of lizard patterns varied with size (SVL) for all observers. At close viewing distances, lizards are predicted to see more distinct patches in the dorsal patterns of other lizards, while their avian predators are predicted to see a reduction in pattern diversity and complexity as lizards get larger. The number of clusters reduces substantially at greater viewing distances for both predators; at such distances, size no longer has a strong impact on dorsal pattern information (other than a slight increase in the number of clusters with increased size for butcherbirds). Body size is an important mediator of predator success and thus an influential component of the selective pressure faced by prey species. It is therefore not surprising that dorsal patterns vary with body size. Indeed, comparative analyses reveal that the employment of various camouflage strategies is influenced by the relative body size of species (Cavagnaro et al. 2023; Caro and Koneru 2021), while studies of individuals within a species indicated that the differential effectiveness of background matching is related to body size (Barnett et al. 2023). We have not quantified background matching in this study, so the relative effectiveness of patterns for small and large lizards is unclear. In structurally diverse and complex microhabitats, substrate selection might be particularly important and might drive lizards of different sizes to utilise different microhabitats that are better suited to their appearance. Barnett et al. (2023) also hypothesise that substrate selection might be driven by body size in *Anaxyrus fowleri* toads, as their spot patterns scale within individuals as they age (and grow). Further work is needed to determine whether the lizard patterns we investigated change during ontogeny in a similar manner and whether between‐observer differences are ecologically relevant.

Our results point to differences in dorsal patterns between males and females, but the nature of the differences is viewer and distance dependent. Lizard observers at close viewing distances would perceive males to have more distinct patches, higher colour contrast and overall luminance than females. In contrast, kookaburras are expected to perceive males to have lower colour contrast compared with females. Sex differences in visual appearance are common in lizard species (e.g., Phrynosomatidae: Stephenson and Ramírez‐Bautista 2012; Anoles: Butler et al. 2007; Teiidae: Anderson and Vitt 1990), while Jacky dragons exhibit dimorphism in morphological traits. The present data provide further evidence that visual appearance might provide additional cues to sexual identity in this species. Disparity in the direction of the difference is intriguing and probably reflects cone sensitivity profiles of the different observers, but dorsal patterns are probably not relevant during close‐distance interactions between lizards and kookaburras. Appearance to avian predators at greater viewing distances is more biologically relevant, and at such distances avian predators see greater diversity and complexity in male patterns with higher overall luminance and colour values. As we have not quantified conspicuousness, we do not know whether this offers an advantage or disadvantage to males. Male Jacky lizards do not select perch sites randomly (Salisbury and Peters 2019), so the biological significance of sex differences in appearance in the context of microhabitat selection warrants further investigation (Recknagel et al. 2023).

While appearance might influence microhabitat selection, our results suggest influence is not in one direction, as the local environment is also a contributor to intraspecific variation in visual appearance in Jacky dragons. Local adaptation of lizard colouration has been reported previously (Rosenblum 2006; McLean et al. 2014; Marshall et al. 2015), and there is evidence for other *A. muricatus* traits varying geographically (Barquero et al. 2015; Ramos and Peters 2017) and for elements of their patterning to vary temporally (Raynal et al. 2022). Our results show an effect of habitat for both lizard and avian predator observers. While the specifics of these differences are observer dependent, the general pattern is for greater diversity and complexity in lizards that are in coastal heath environments. Relative to dry open forest and rocky outcroppings that Jacky dragons occupy at inland locations, we typically regard coastal heathlands to represent a more visually complex landscape (Ramos and Peters 2017). Additional work is required to quantify the diversity of microhabitat options, but it is possible that greater complexity in patterns is driven by the heterogeneity of the local environment (Merilaita 2007).

Comparisons between different colour morphs within a species have helped elucidate the genetics underpinning colouration (Cuthill et al. 2017). Studies of the genetic component of colour and patterns in species not characterised by discrete morphs are less common but include recent work demonstrating that aspects of dorsal patterns of Jacky dragons are heritable in a pedigree lab population sourced from a single location (Raynal et al. 2022). Complementing these findings, our results show phenotypic divergence in appearance between multiple populations as a function of genetic divergence between clades, and such differences are detectable by both conspecifics and avian predators. Although each of the four clades differed from each of the other clades in some way, multiple significant differences between lizards from clades B and C, and to a lesser extent clades A and C, were reported. The latter is to be expected as the environments are very different, but consistent differences between clades B and C were not anticipated. Lizards from clade B are all coastal heath lizards, as are most (93%) of the lizards sampled from clade C. Heritability values in Raynal et al. (2022) are lower than in other similar studies, and one explanation points to important contributions from non‐genetic drivers of variation. In contrast, Gervais et al. (2022) argue that heritability values are often inflated when individuals share the same environments. Environmental drivers of phenotypic appearance are probably relevant, as we suggest above, but our data imply that genetic influence could maintain pattern diversity when the local environmental influence is not strong enough to promote convergence in pattern detail. An alternative explanation is that assuming environmental similarity based on broad‐scale vegetation classification is misleading and that dorsal patterns of these lizards respond to the interaction of multiple ecological circumstances. One ecological component that might vary between populations within the respective clades is the relative abundance of avian predators, and our findings suggest that observer identity might be crucial.

Avian predators in general have been implicated as significant influences on many traits in lizards (reviewed in Blomberg and Shine 2000). However, the results we presented herein provide evidence that kookaburras and butcherbirds may vary in their ability to detect these lizards as prey, and it is therefore likely that the predation threat they represent to the lizards likewise varies. While we do not explicitly assess conspicuousness, the quantified pattern features we have measured are relevant to early visual processes for edge detection and object identification, and hence very pertinent to predation avoidance (Endler 2012). Kookaburras are known to be sit‐and‐wait ambush predators, often found perched in the lower branches of trees with craned heads looking for prey (Legge 2002). Detection of prey in this manner could be heavily reliant on movement for detection (Allen et al. 2009) and only reliably recognise motionless prey against unsuitable backgrounds. In contrast, grey butcherbirds do employ elevated scanning for predation but also engage in active hunting for airborne insects, birds and nests (Jasper 1963; Greig et al. 2010), which may indicate they are more oriented towards detection and tracking of rapid movements from closer distances and so could represent a significantly different pressure on the phenotypes of Jacky dragon camouflage. Both kookaburras and butcherbirds are likely to be present in all tested habitat types but may occur in different densities and thus represent varying threats. Predation threat also varies where predators are familiar with prey species, or the presence and density of other prey species varies (Stuart‐Fox et al. 2003).

By quantifying dorsal patterns through the specific visual constraints of relevant observers, we demonstrate that Jacky dragon dorsal appearance varies not only objectively but also in ways that are functionally meaningful to both conspecifics and potential predators. While variation in pattern and observer perception has been studied in many species, our findings highlight fine‐scale differences that would likely be overlooked if either factor were examined in isolation. Although animal colouration is shaped by local environmental conditions, it is clearly the product of complex interactions among ecological needs, habitat characteristics and predation risk. Beyond the avenues for future research already discussed, other factors merit further study. Colouration may respond to short‐term environmental fluctuations (e.g., changes in food availability, vegetation or predator presence), and some degree of developmental or behavioural flexibility (Stevens and Ruxton 2019) could be advantageous. Moreover, physiological shifts in colour or contrast related to thermoregulation (Wuthrich et al. 2022) may affect how Jacky dragons are perceived by other organisms and should be investigated further.

## Author Contributions

**Jonathan W. Salisbury:** conceptualization (equal), data curation (lead), formal analysis (lead), funding acquisition (supporting), investigation (lead), methodology (lead), project administration (lead), validation (equal), visualization (equal), writing – original draft (lead), writing – review and editing (equal). **Richard A. Peters:** conceptualization (equal), data curation (supporting), formal analysis (supporting), funding acquisition (lead), investigation (supporting), methodology (supporting), project administration (supporting), supervision (lead), validation (equal), visualization (equal), writing – original draft (supporting), writing – review and editing (equal).

## Ethics Statement

All data collection was undertaken in accordance with the ethical policies of La Trobe University (AEC 17–57) and under permits provided by the Department of Environment, Land, Water and Planning in Victoria (No. 10008503; Import Permit No. 14641671) and the National Parks & Wildlife Service of the Office of Environment & Heritage in New South Wales (No. SL101987).

## Conflicts of Interest

The authors declare no conflicts of interest.

## Supporting information


**Appendix S1:** ece371944‐sup‐0001‐AppendixS1.pdf.

## Data Availability

Original data and R script files are available at La Trobe University's FigShare account and can be accessed during review here: https://figshare.com/s/4b8b3e1af1c4ae5e8a2c. This private link will be replaced with a DOI at the time of publication.

## Appendix A

**TABLE A1:** Summary of notable output from QCPA analysis of lizard dorsal patterns, after visual limitations of modelled observer are applied.

Output	Name	Description
Clusters		Number of discreet patches of homogenous colour
Counts		Numbers of distinct patches within each cluster
CAA:Pt	Patch size	Measure of average size of each patch
*CCA:Sc*	*Simpson colour diversity*	*Measure of how evenly colours are represented*
CAA:Jc	Relative Simpson colour diversity	Measure of how evenly colours are represented independent of the number of colours
CAA:Hc	Shannon colour diversity	Alternate measure of colour diversity
CAA:Ht	Shannon transition diversity	Measure of regularity of transitions between different colours
CAA:C	Pattern complexity	Measure of pattern complexity based on transition frequencies
VCA:ML	Pattern luminance contrast	Weighted mean luminance of the image
VCA:MDmax	Pattern Dmax contrast	Weighted mean of Dmax contrast of the image weighted by the area of each colour
VCA:MSsat	Pattern RNL saturation contrast	Weighted mean RNL saturation of the image weighted by the area of each colour
BSA:BML	Luminance boundary strength	Weighted mean luminance difference of boundaries between colours
BSA:BMDmax	Dmax boundary strength	Weighted mean Dmax chromaticity difference of boundaries between colours
BSA:BMSsat	RNL saturation boundary strength	The weighted mean RNL saturation difference of boundaries between colours
BSA:BMSL	RNL luminance boundary strength	Weighted mean RNL luminance difference of boundaries between colours
BSA:BMS	RNL chromaticity boundary strength	Weighted mean RNL colour difference of boundaries between colours

*Note:* See van den Berg et al. (2020) for full list and details of outputs.

## Appendix B

**TABLE B1:** Variable loadings from PCA for each observer at 1 and 5 m observation distances.

Variable	1 m observation distance			5 m observation distance		
	PC1	PC2	PC3	PC1	PC2	PC3
Lizards						
CAA.Sc	−0.334	−0.057	0.290			
CAA.Jc	0.222	−0.070	0.030			
CAA.Hc	−0.378	−0.029	0.254			
CAA.Ht	−0.336	−0.061	0.287			
CAA.C	−0.342	−0.030	0.203			
VCA.ML	0.001	−0.374	0.303			
VCA.MDmax	0.010	0.623	0.078			
VCA.MSsat	0.020	0.624	0.048			
BSA.BML	−0.310	0.132	0.062			
BSA.BMDmax	−0.268	−0.156	−0.520			
BSA.BMSsat	−0.300	−0.026	−0.516			
BSA.BMSL	−0.309	0.131	0.068			
BSA.BMS	−0.336	0.095	−0.287			
Butcherbirds						
CAA.Sc	−0.310	0.325	−0.028	−0.343	0.001	0.087
CAA.Jc	0.128	0.214	0.103	0.240	0.128	0.213
CAA.Hc	−0.362	0.281	−0.026	−0.376	−0.052	0.110
CAA.Ht	−0.365	0.270	−0.100	−0.349	−0.008	0.020
CAA.C	−0.340	0.231	0.127	−0.320	−0.049	0.145
VCA.ML	−0.053	−0.158	−0.226	−0.016	0.419	0.461
VCA.MDmax	0.225	0.461	0.184	0.141	−0.607	0.129
VCA.MSsat	0.203	0.450	0.257	0.147	−0.596	0.108
BSA.BML	0.174	0.229	−0.623	−0.278	−0.084	0.393
BSA.BMDmax	−0.368	−0.245	−0.113	−0.301	0.061	−0.426
BSA.BMSsat	−0.330	−0.103	−0.136	−0.283	−0.028	−0.422
BSA.BMSL	0.169	0.231	−0.626	−0.275	−0.081	0.396
BSA.BMS	−0.331	0.164	0.009	−0.307	−0.247	−0.057
Kookaburra						
CAA.Sc	0.362	0.117	−0.169	−0.316	0.291	−0.073
CAA.Jc	−0.221	0.168	0.037	0.218	0.132	−0.484
CAA.Hc	0.400	0.086	−0.195	−0.357	0.137	−0.008
CAA.Ht	0.408	0.088	−0.170	−0.291	0.213	0.259
CAA.C	0.357	0.104	−0.113	−0.342	0.099	−0.119
VCA.ML	0.074	−0.286	−0.147	−0.029	−0.074	0.697
VCA.MDmax	0.208	−0.432	−0.145	−0.203	0.414	−0.023
VCA.MSsat	0.177	−0.524	−0.191	−0.156	0.495	−0.072
BSA.BML	−0.121	0.327	−0.535	−0.323	−0.232	0.115
BSA.BMDmax	0.288	0.260	0.281	−0.243	−0.377	−0.202
BSA.BMSsat	0.238	0.325	0.385	−0.262	−0.384	−0.267
BSA.BMSL	−0.115	0.326	−0.538	−0.322	−0.231	0.122
BSA.BMS	0.346	0.046	0.094	−0.347	−0.072	−0.218

## Appendix C

**TABLE C1:** Pairwise contrasts for lizard observers at 1 m.

Contrast	Estimate	SE	df	*z*	*p*
Number of clusters					
A‐B	−0.118	0.353	Inf	−0.334	1.000
A‐C	0.495	0.408	Inf	1.212	0.784
A‐D	0.104	0.256	Inf	0.408	0.999
B‐C	0.613	0.213	Inf	2.875	0.024
B‐D	0.222	0.403	Inf	0.551	0.995
C‐D	−0.390	0.458	Inf	−0.852	0.951
Counts within clusters					
A‐B	−0.099	0.273	Inf	−0.363	1.000
A‐C	0.550	0.317	Inf	1.736	0.403
A‐D	0.180	0.204	Inf	0.883	0.942
B‐C	0.649	0.166	Inf	3.917	0.001
B‐D	0.279	0.316	Inf	0.882	0.942
C‐D	−0.370	0.359	Inf	−1.033	0.884
PC2 score					
A‐B	−1.400	0.519	53	−2.702	0.054
A‐C	−3.650	0.777	53	−4.705	0.000
A‐D	−2.480	0.654	53	−3.788	0.002
B‐C	−2.250	0.556	53	−4.050	0.001
B‐D	−1.080	0.660	53	−1.628	0.501
C‐D	1.180	0.888	53	1.327	0.718
PC3 score—comparing habitat types within sex					
Males	−2.52	0.675	52	−3.74	0.0005
Females	−1.59	0.708	52	−2.247	0.0289

**TABLE C2:** Pairwise contrasts between habitat types within sex for butcherbird observers at 1 m.

Variable	Sex	Estimate	SE	df	*z*.ratio	*p*
Clusters	Males	1.327	0.289	Inf	4.587	< 0.0001
	Females	0.281	0.272	Inf	1.034	0.301
Counts	Males	1.640	0.167	Inf	9.822	< 0.0001
	Females	1.700	0.172	Inf	9.874	< 0.0001
Colour	Males	−0.012	0.055	49	−0.217	0.829
	Females	−0.001	0.060	49	−0.025	0.981

**TABLE C3:** Pairwise contrasts for butcherbird observers at 1 m.

Contrast	Estimate	SE	df	*z*.ratio	*p*
PC1 score					
A‐B	−0.514	0.897	53	−0.573	0.994
A‐C	−3.195	1.343	53	−2.379	0.120
A‐D	−1.950	1.130	53	−1.725	0.434
B‐C	−2.681	0.962	53	−2.787	0.043
B‐D	−1.436	1.142	53	−1.258	0.764
C‐D	1.245	1.535	53	0.811	0.962
PC2 score					
A‐B	−1.060	0.571	53	−1.856	0.349
A‐C	−2.297	0.855	53	−2.685	0.057
A‐D	−2.979	0.720	53	−4.138	0.001
B‐C	−1.237	0.613	53	−2.019	0.258
B‐D	−1.920	0.727	53	−2.640	0.064
C‐D	−0.682	0.978	53	−0.698	0.982
Clusters—males					
A‐B	0.828	0.284	Inf	2.916	0.0211
A‐C	1.492	0.299	Inf	4.982	< 0.0001
A‐D	0.399	0.127	Inf	3.146	0.0099
B‐C	0.664	0.103	Inf	6.477	< 0.0001
B‐D	−0.429	0.299	Inf	−1.434	0.627
C‐D	−1.093	0.316	Inf	−3.46	0.0032
Clusters—females					
A‐B	0.705	0.249	Inf	2.828	0.0278
A‐C	1.092	0.287	Inf	3.808	0.0008
A‐D	−0.236	0.163	Inf	−1.448	0.6166
B‐C	0.387	0.141	Inf	2.744	0.0358
B‐D	−0.941	0.287	Inf	−3.279	0.0062
C‐D	−1.328	0.321	Inf	−4.143	0.0002
Counts—males					
A‐B	1.282	0.1646	Inf	7.79	< 0.0001
A‐C	1.619	0.1707	Inf	9.486	< 0.0001
A‐D	0.356	0.0578	Inf	6.158	< 0.0001
B‐C	0.337	0.0484	Inf	6.971	< 0.0001
B‐D	−0.926	0.17	Inf	−5.449	< 0.0001
C‐D	−1.264	0.1767	Inf	−7.152	< 0.0001
Counts—females					
A‐B	1.824	0.1682	Inf	10.846	< 0.0001
A‐C	2.49	0.1755	Inf	14.183	< 0.0001
A‐D	−0.36	0.0613	Inf	−5.879	< 0.0001
B‐C	0.665	0.0499	Inf	13.337	< 0.0001
B‐D	−2.184	0.1762	Inf	−12.397	< 0.0001
C‐D	−2.85	0.1834	Inf	−15.538	< 0.0001

**TABLE C4:** Pairwise contrasts for kookaburra observers at 1 m.

Contrast	Estimate	SE	df	*z*.ratio	*p*
PC1 score					
A‐B	0.554	0.569	53	0.974	0.913
A‐C	−1.167	0.852	53	−1.371	0.688
A‐D	−1.679	0.717	53	−2.341	0.130
B‐C	−1.721	0.610	53	−2.821	0.040
B‐D	−2.232	0.724	53	−3.083	0.019
C‐D	−0.511	0.973	53	−0.525	0.996
PC2 score					
A‐B	0.554	0.569	53	0.974	0.913
A‐C	−1.167	0.852	53	−1.371	0.688
A‐D	−1.679	0.717	53	−2.341	0.130
B‐C	−1.721	0.610	53	−2.821	0.040
B‐D	−2.232	0.724	53	−3.083	0.019
C‐D	−0.511	0.973	53	−0.525	0.996
Counts—males					
A‐B	0.655	0.108	Inf	6.047	< 0.0001
A‐C	0.645	0.119	Inf	5.422	< 0.0001
A‐D	0.504	0.055	Inf	9.258	< 0.0001
B‐C	−0.010	0.052	Inf	−0.194	1.000
B‐D	−0.151	0.116	Inf	−1.298	0.727
C‐D	−0.141	0.127	Inf	−1.107	0.846
Counts—females					
A‐B	1.912	0.159	Inf	11.989	< 0.0001
A‐C	2.226	0.169	Inf	13.148	< 0.0001
A‐D	0.021	0.066	Inf	0.315	1.000
B‐C	0.315	0.057	Inf	5.547	< 0.0001
B‐D	−1.891	0.170	Inf	−11.102	< 0.0001
C‐D	−2.205	0.180	Inf	−12.270	< 0.0001

**TABLE C5:** Contrast between habitat types within sex for kookaburra observers at 1 m.

Variable	Sex	Estimate	SE	df	*z*.ratio	*p*
Counts	Males	0.557	0.115	Inf	4.843	< 0.0001
	Females	1.240	0.166	Inf	7.481	< 0.0001
Colour	Males	−0.009	0.042	52	−0.224	0.823
	Females	−0.088	0.044	52	−1.982	0.053

## Appendix D

**TABLE D1:** Pairwise contrasts between clades for butcherbird observers at 5 m.

Contrast	Estimate	SE	df	*z*.ratio	*p*
Clusters					
A‐B	0.205	0.394	Inf	0.520	0.996
A‐C	0.758	0.447	Inf	1.698	0.430
A‐D	0.289	0.265	Inf	1.092	0.855
B‐C	0.554	0.216	Inf	2.564	0.061
B‐D	0.084	0.447	Inf	0.188	1.000
C‐D	−0.469	0.498	Inf	−0.942	0.922
Counts					
A‐B	0.272	0.314	Inf	0.868	0.946
A‐C	1.029	0.358	Inf	2.871	0.024
A‐D	0.365	0.211	Inf	1.731	0.407
B‐C	0.756	0.179	Inf	4.217	0.000
B‐D	0.093	0.357	Inf	0.260	1.000
C‐D	−0.663	0.401	Inf	−1.653	0.463
PC1 score					
A‐B	−1.256	0.899	53	−1.397	0.669
A‐C	−4.237	1.347	53	−3.145	0.016
A‐D	−1.504	1.134	53	−1.326	0.719
B‐C	−2.981	0.965	53	−3.089	0.019
B‐D	−0.248	1.145	53	−0.216	1.000
C‐D	2.733	1.540	53	1.775	0.400
PC2 score					
A‐B	1.508	0.467	53	3.231	0.013
A‐C	3.382	0.699	53	4.837	0.000
A‐D	2.809	0.589	53	4.773	0.000
B‐C	1.873	0.501	53	3.741	0.003
B‐D	1.301	0.594	53	2.188	0.183
C‐D	−0.573	0.799	53	−0.717	0.980

**TABLE D2:** Pairwise contrasts for kookaburra observers at 5 m.

Contrast	Estimate	SE	df	*z*.ratio	*p*
Clusters					
A‐B	−0.057	0.399	Inf	−0.142	1.000
A‐C	0.609	0.454	Inf	1.341	0.696
A‐D	0.300	0.300	Inf	1.001	0.898
B‐C	0.666	0.227	Inf	2.931	0.020
B‐D	0.357	0.465	Inf	0.768	0.970
C‐D	−0.309	0.519	Inf	−0.595	0.992
Counts					
A‐B	0.055	0.303	Inf	0.182	1.000
A‐C	0.608	0.348	Inf	1.746	0.397
A‐D	0.128	0.213	Inf	0.599	0.992
B‐C	0.553	0.178	Inf	3.112	0.011
B‐D	0.073	0.347	Inf	0.210	1.000
C‐D	−0.480	0.392	Inf	−1.224	0.776
Counts—compare habitat types within sex					
Males	0.5205	0.336	Inf	1.547	0.1219
Females	−0.0275	0.35	Inf	−0.079	0.9374
