# Supplementary material for: Intraspecific Variation in Dorsal Colour Patterns of Amphibolurus muricatus Lizards From the Perspective of Relevant Observers
Source: Ecol Evol. 2025 Aug 11;15(8):e71944. doi: 10.1002/ece3.71944 (PMC12339052; doi:10.1002/ece3.71944)
Supplement: Supplementary file 1 — Appendix S1: ece371944‐sup‐0001‐AppendixS1.pdf. [file ECE3-15-e71944-s001.pdf]

SUPPLEMENTARY MATERIAL

Accompanying

Laughing kookaburra, hidden dragon: dorsal colour patterns and intraspecific variation of *Amphibolurus muricatus* from the perspective of relevant observers

Jonathan W. Salisbury[1,2] and Richard A Peters[1,2,3]

[1] Animal Behaviour Group, La Trobe University, Melbourne, VIC, Australia

[2] Department of Ecological, Plant and Animal Sciences, School of Agriculture, Biomedicine & Environment, La Trobe University, Melbourne, VIC, Australia

[3] Corresponding author

Richard Peters
Discipline of Animal Biology & Health
Department of Ecological, Plant and Animal Sciences
La Trobe University
Bundoora, Victoria, 3086
Australia
richard.peters@latrobe.edu.au
+61 3 9489 2234

Comparison between observers

Methods - Pattern variation between observers

The dorsal region of interest (ROI) for each lizard pattern was extracted as described in the main text and analysed multiple times from the perspective of different observers and viewing distances. The visual capabilities of four potential observers of the lizards were used and are defined by cone types and their relative absorption profiles, as well as visual acuity values (cycles per degree, cpd). We modelled the visual capabilities of humans (*Homo sapiens*), Jacky dragons (*A. muricatus*), grey butcherbirds (*Cracticus torquatus*) and laughing kookaburras (*Dacelo novaguineae*). Spectral sensitivities of lizards and avian predators are outlined in Figure S1. To these we added spectral sensitivities of humans, which typically possess three cone types with peak sensitivities of 420-440 nm, 530-540 nm and 560-580 nm and and spatial acuity of 72 cpd. We followed the general approach described in the main text to summarise dorsal patterns in terms of cluster statistics, local edge intensity as well as multiple colour pattern measurements including colour adjacency analysis (CAA), boundary strength analysis (BSA) and visual contrast analysis (VCA).

We selected all output at a viewing distance of 1 m and constructed generalised linear models (*glm* function) to examine variation in the dependent variable as a function of observer (4 levels: lizard, butcherbird, kookaburra, human) and snout-vent length (SVL). The number of clusters and counts within clusters were examined using a Poisson distribution and a log link function. Models examining the mean colour intensity and luminance intensity were fitted with gaussian distributions but required square-root transformation of the response variables. We selected three colour pattern variables to examine: colour pattern complexity (CAA:C variable from QCPA) where higher values reflect more complex patterns, and measures of luminance and chromaticity boundary strength (BSA:BMSL and BSA:BMS respectively) where higher values reflect higher luminance/colour differences across boundaries. Models constructed to examine colour pattern variables were fitted with gaussian distributions. The significance of all models was assessed using the *Anova* function from the *car* package (Fox & Weisberg 2019) where the main effects of observer and SVL are computed from Type II sums of squares. We repeated the above set of analyses after selecting output obtained using a viewing distance of 5 m.

Results - Pattern variation between observers

Detectable pattern features and details did vary between observers at both 1 m and 5 m for all variables measured (Table S1). Pairwise contrasts adjusted for the number of tests to determine differences between the observers are reported in Tables S2 and S3). At 1 m viewing distance butcherbirds detect more clusters and counts within those clusters than kookaburras, although both avian observers measured higher than lizards or humans (Figure S2a,b). At 5 m clusters/counts were lower overall, and butcherbirds and kookaburras showed no difference in this measure, though both were still higher than lizards and clusters was higher than humans (Figure S2e,f). Luminance intensity at 1 m showed no difference between avian predators, with lizards highest and humans lowest (Figure S2c). At 5 m lizards recorded much lower mean intensity, lower than both birds and humans (Figure S2g). Butcherbirds observed the highest levels of colour intensity at 1 m, followed by kookaburras, then lizards and humans (Figure S2d). At 5 m this pattern repeated, though at lower overall levels and with less discernible difference between lizard and humans (Figure S2h). Functional pattern colour complexity was highest for butcherbirds, above kookaburras at both 1 m and 5 m, while humans were higher than lizards at 1 m, but lower at 5 m (Figure S3a,d). Analysis of boundary strength for luminance and colour again showed significant differences between observers. Butcherbirds had the highest values for colour boundary strength at 1 m, followed by kookaburras, lizards and then humans (Figure S3b), with a similar pattern at 5 m (Figure S3e). Lizards measured above both birds but below humans for luminance at 1 m (Figure S3c), but well below all the other observers at 5 m (Figure S3f).

Table S1: Statistical outcomes for testing effect of observer on features of lizard camouflage pattern. Observers modelled Jacky dragons (*Amphibolurus muricatus*), laughing kookaburra (*Dacelo novaguineae*), grey butcherbird (*Cracticus torquatus*) and Humans.

Terms	SS Type	Df	Test statistic	Residual Df	Residual Dev.	P-value
			1 m viewing distance			
Clusters						
**Observer**	**I**	**3**	**1883.87**	**236**	**655.21**	**<0.001**
**SVL**	**I**	**1**	**4.72**	**235**	**650.49**	**0.030**
Counts						
**Observer**	**I**	**3**	**13207.6**	**236**	**11439**	**<0.001**
SVL	I	1	1.8	235	11438	0.177
Colour mean						
**Observer**	**I**	**3**	**2.515**	**236**	**1.096**	**<0.001**
**SVL**	**I**	**1**	**0.0327**	**235**	**1.064**	**0.007**
Luminance						
**Observer**	**I**	**3**	**0.494**	**236**	**1.121**	**<0.001**
**SVL**	**I**	**1**	**0.0367**	**235**	**1.085**	**0.005**
CAA.C						
**Observer**	**I**	**3**	**0.075**	**236**	**0.096**	**<0.001**
**SVL**	**I**	**1**	**0.003**	**235**	**0.093**	**0.003**
BSA.BMSL						
**Observer**	**I**	**3**	**144.29**	**236**	**607.12**	**<0.001**
SVL	I	1	8.50	235	598.62	0.068
			5 m viewing distance			
Clusters						
**Observer**	**I**	**3**	**110.665**	**236**	**152.20**	**<0.001**
**SVL**	**I**	**1**	**6.174**	**235**	**146.02**	**0.013**
Counts						
**Observer**	**I**	**3**	**277.186**	**236**	**503.45**	**<0.001**
**SVL**	**I**	**1**	**5.016**	**235**	**498.43**	**0.025**
Colour mean						
**Observer**	**I**	**3**	**1.717**	**236**	**1.093**	**<0.001**
SVL	I	1	0.001	235	1.092	0.717
Luminance						
**Observer**	**I**	**3**	**0.328**	**236**	**1.095**	**<0.001**
SVL	I	1	0.005	235	1.090	0.282
CAA.C						
**Observer**	**I**	**3**	**0.070**	**236**	**0.099**	**<0.001**
SVL	I	1	0.00001	235	0.099	0.871
BSA.BMSL						
**Observer**	**I**	**3**	**583.01**	**236**	**973.19**	**<0.001**
**SVL**	**I**	**1**	**32.04**	**235**	**941.15**	**0.005**

Table S2: Pairwise comparisons for between observer effects at a viewing distance of 1 m

Variable	contrast	estimate	SE	df	z.ratio	p.value
Number of clusters	Li-Bb	-1.960	0.0720	Inf	-27.219	<.0001
	Li-Kb	-0.799	0.0812	Inf	-9.839	<.0001
	Li-Hu	0.370	0.1055	Inf	3.506	0.0027
	Bb-Kb	1.161	0.0518	Inf	22.412	<.0001
	Bb-Hu	2.330	0.0850	Inf	27.421	<.0001
	Kb-Hu	1.168	0.0929	Inf	12.583	<.0001
Number of counts	Li-Bb	-3.211	0.0538	Inf	-59.736	<.0001
	Li-Kb	-3.316	0.0537	Inf	-61.809	<.0001
	Li-Hu	-2.462	0.0549	Inf	-44.840	<.0001
	Bb-Kb	-0.105	0.0146	Inf	-7.200	<.0001
	Bb-Hu	0.749	0.0187	Inf	40.115	<.0001
	Kb-Hu	0.854	0.0184	Inf	46.488	<.0001
Colour intensity	Li-Bb	-0.1230	0.0123	235	-10.016	<.0001
	Li-Kb	-0.0527	0.0123	235	-4.290	0.0002
	Li-Hu	0.1553	0.0123	235	12.640	<.0001
	Bb-Kb	0.0703	0.0123	235	5.725	<.0001
	Bb-Hu	0.2783	0.0123	235	22.656	<.0001
	Kb-Hu	0.2080	0.0123	235	16.930	<.0001
Luminance intensity	Li-Bb	0.0328	0.0124	235	2.643	0.0515
	Li-Kb	0.0439	0.0124	235	3.538	0.0029
	Li-Hu	0.1235	0.0124	235	9.955	<.0001
	Bb-Kb	0.0111	0.0124	235	0.895	0.9385
	Bb-Hu	0.0907	0.0124	235	7.311	<.0001
	Kb-Hu	0.0796	0.0124	235	6.417	<.0001
Colour complexity (CAA)	Li-Bb	-0.02353	0.00363	235	-6.478	<.0001
	Li-Kb	-0.00985	0.00363	235	-2.711	0.0424
	Li-Hu	0.02487	0.00363	235	6.848	<.0001
	Bb-Kb	0.01368	0.00363	235	3.766	0.0013
	Bb-Hu	0.04840	0.00363	235	13.325	<.0001
	Kb-Hu	0.03472	0.00363	235	9.559	<.0001
Boundary strength (colour)	Li-Bb	-1.028	0.106	235	-9.663	<.0001
	Li-Kb	-0.451	0.106	235	-4.237	0.0002
	Li-Hu	1.199	0.106	235	11.268	<.0001
	Bb-Kb	0.577	0.106	235	5.426	<.0001
	Bb-Hu	2.227	0.106	235	20.931	<.0001
	Kb-Hu	1.650	0.106	235	15.505	<.0001
Boundary strength (luminance)	Li-Bb	0.650	0.291	235	2.231	0.1496
	Li-Kb	0.201	0.291	235	0.689	0.9827
	Li-Hu	-1.423	0.291	235	-4.882	<.0001
	Bb-Kb	-0.449	0.291	235	-1.542	0.5497
	Bb-Hu	-2.073	0.291	235	-7.113	<.0001
	Kb-Hu	-1.623	0.291	235	-5.571	<.0001

Table S3: Pairwise comparisons for between observer effects at a viewing distance of 5 m

Variable	contrast	estimate	SE	df	z.ratio	p.value
Number of clusters	Li-Bb	-1.269	0.1449	Inf	-8.757	<.0001
	Li-Kb	-1.115	0.1475	Inf	-7.556	<.0001
	Li-Hu	-0.634	0.1584	Inf	-4.003	0.0004
	Bb-Kb	0.154	0.0999	Inf	1.543	0.5447
	Bb-Hu	0.635	0.1153	Inf	5.505	<.0001
	Kb-Hu	0.481	0.1186	Inf	4.053	0.0003
Number of counts	Li-Bb	-1.694	0.1393	Inf	-12.162	<.0001
	Li-Kb	-1.703	0.1392	Inf	-12.235	<.0001
	Li-Hu	-1.694	0.1393	Inf	-12.162	<.0001
	Bb-Kb	-0.009	0.0774	Inf	-0.116	1.0000
	Bb-Hu	0.000	0.0776	Inf	0.000	1.0000
	Kb-Hu	0.009	0.0774	Inf	0.116	1.0000
Colour intensity	Li-Bb	-1.90e-01	0.0124	235	-15.296	<.0001
	Li-Kb	-1.40e-01	0.0124	235	-11.288	<.0001
	Li-Hu	9.53e-05	0.0124	235	0.008	1.0000
	Bb-Kb	4.99e-02	0.0124	235	4.008	0.0005
	Bb-Hu	1.90e-01	0.0124	235	15.304	<.0001
	Kb-Hu	1.41e-01	0.0124	235	11.296	<.0001
Luminance intensity	Li-Bb	-0.08471	0.0124	235	-6.812	<.0001
	Li-Kb	-0.09430	0.0124	235	-7.584	<.0001
	Li-Hu	-0.04897	0.0124	235	-3.938	0.0006
	Bb-Kb	-0.00959	0.0124	235	-0.772	0.9695
	Bb-Hu	0.03574	0.0124	235	2.874	0.0262
	Kb-Hu	0.04533	0.0124	235	3.646	0.0020
Colour complexity (CAA)	Li-Bb	-0.04451	0.00375	235	-11.881	<.0001
	Li-Kb	-0.03687	0.00375	235	-9.842	<.0001
	Li-Hu	-0.02081	0.00375	235	-5.554	<.0001
	Bb-Kb	0.00764	0.00375	235	2.039	0.2298
	Bb-Hu	0.02370	0.00375	235	6.326	<.0001
	Kb-Hu	0.01606	0.00375	235	4.288	0.0002
Boundary strength (colour)	Li-Bb	-1.660	0.13	235	-12.753	<.0001
	Li-Kb	-1.359	0.13	235	-10.439	<.0001
	Li-Hu	-0.234	0.13	235	-1.800	0.3662
	Bb-Kb	0.301	0.13	235	2.314	0.1223
	Bb-Hu	1.426	0.13	235	10.954	<.0001
	Kb-Hu	1.125	0.13	235	8.639	<.0001
Boundary strength (luminance)	Li-Bb	-2.931	0.365	235	-8.022	<.0001
	Li-Kb	-3.648	0.365	235	-9.984	<.0001
	Li-Hu	-3.924	0.365	235	-10.739	<.0001
	Bb-Kb	-0.717	0.365	235	-1.962	0.2695
	Bb-Hu	-0.992	0.365	235	-2.716	0.0418
	Kb-Hu	-0.276	0.365	235	-0.755	0.9727

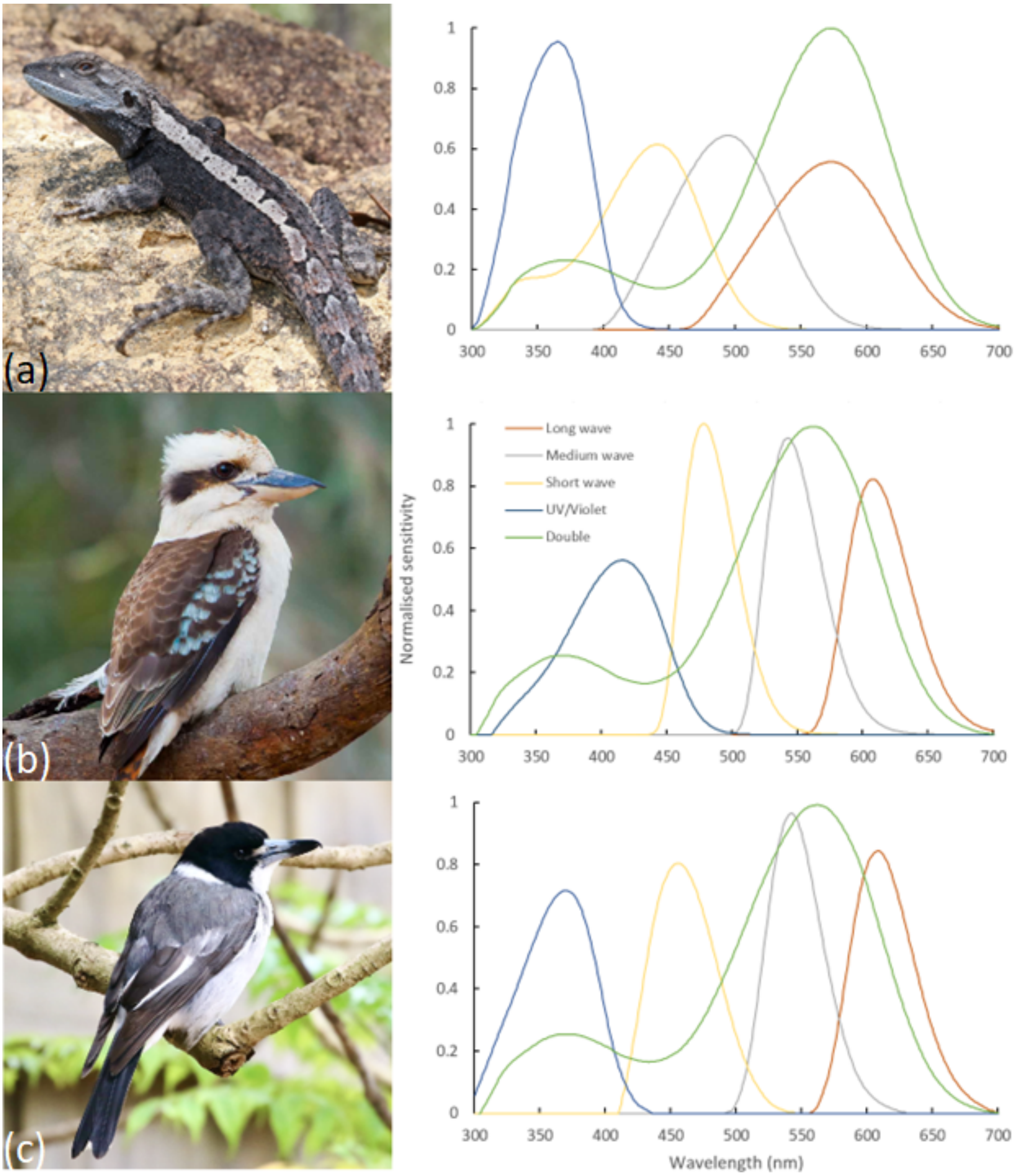

Figure S1: Normalised sensitivity of photoreceptive cones used in models for the eyes of the subject species. (a) Jacky dragons (*Amphibolurus muricatus*) were modelled as: Long wave, λmax = 570nm; Medium wave, λmax = 495nm; Short wave, λmax = 440nm; UV, λmax = 360nm; Double, λmax = 570nm, and relevant potential avian predators based on *Ctenophorus decresii*. The (b) laughing kookaburra (*Dacelo novaguineae)* was specified as: Long wave, λmax = 610nm; Medium wave, λmax = 545nm; Short wave, λmax = 475nm; Violet sensitive, λmax = 415nm; Double, λmax = 560nm. (c) Grey butcherbird *(Cracticus torquatus)* cone sensitivities were set as*:* Long wave, λmax = 610nm; Medium wave, λmax = 540nm; Short wave, λmax = 455nm; UV, λmax = 370nm; Double, λmax = 560nm.

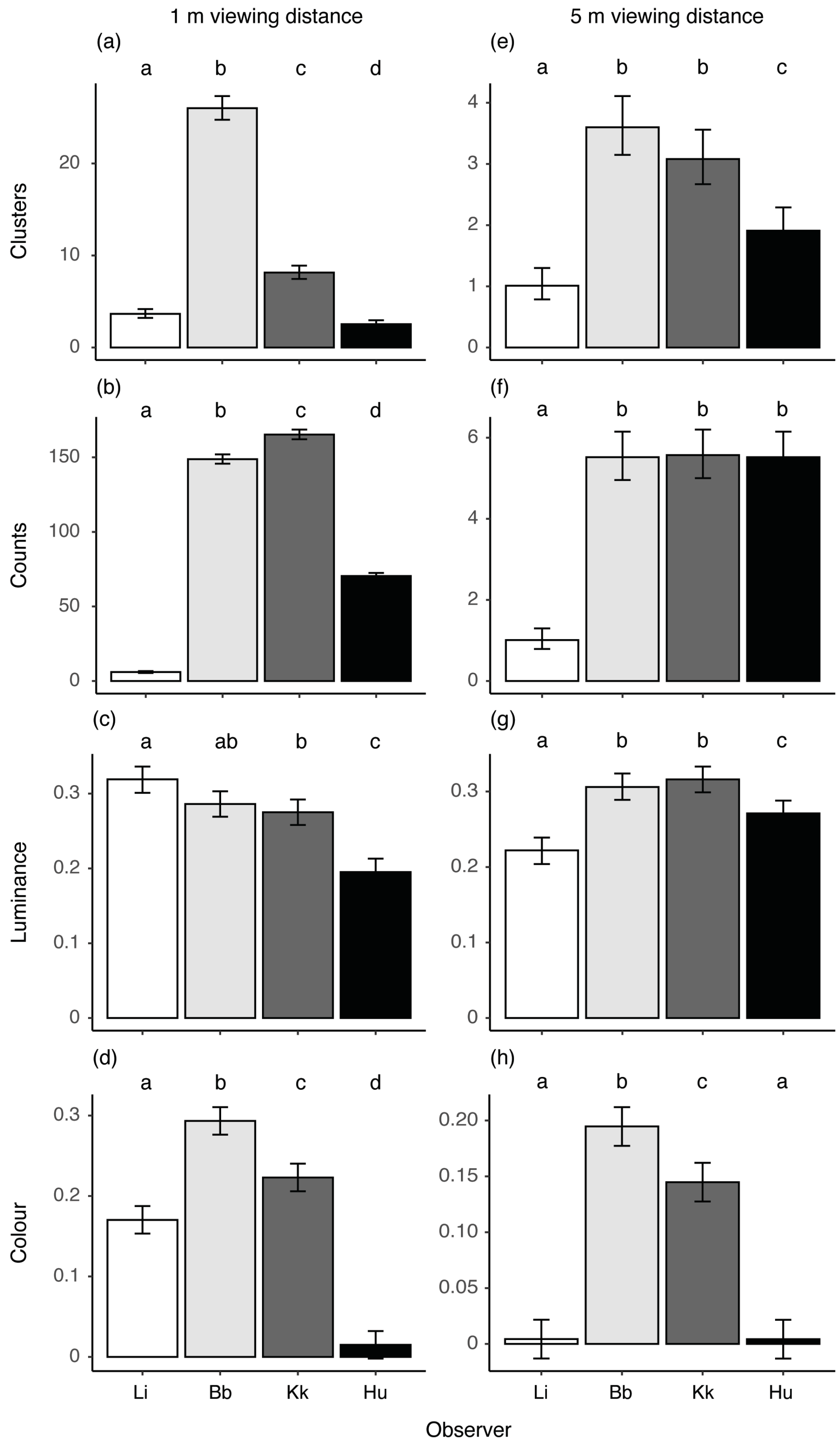

Figure S2: Estimated marginal means predicted from regression models for different observers of Jacky dragon dorsal patterns. Results shown are for (a) the number of clusters, (b) the number of counts within clusters, (c) mean luminance and (d) mean colour at 1 m viewing distance and (e) the number of clusters and (f) counts within clusters, (g) mean luminance and (h) colour at 5 m viewing distance. Observers are lizard (Li), grey butcherbird (Bb), laughing kookaburra (Kb) and humans (Hu). Error bars are 95% CIs. Different letters above bars within plots indicated pairwise differences.

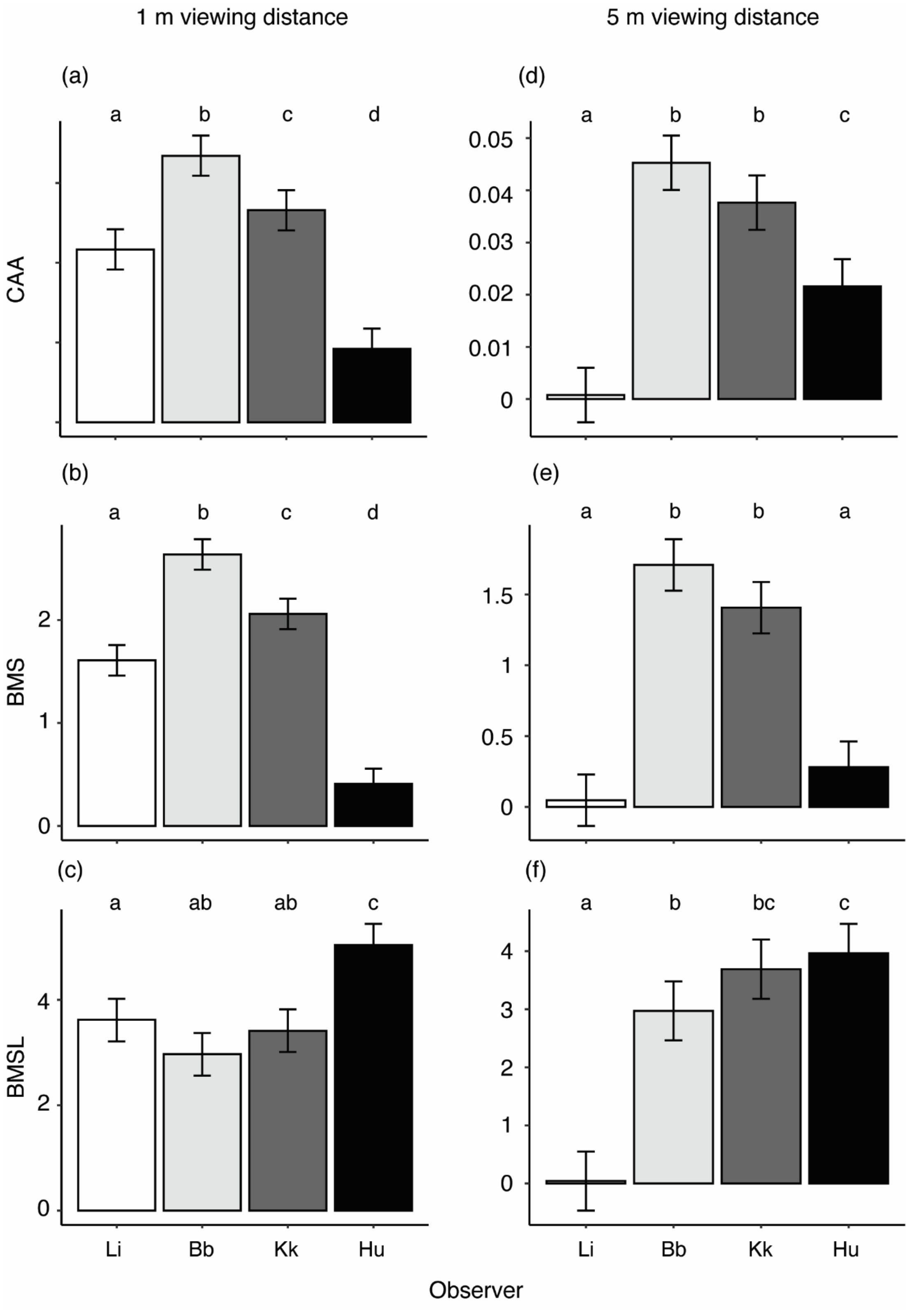

Figure S3: Estimated marginal means predicted from regression models for different observers of Jacky dragon dorsal patterns. Results shown are for (a) colour pattern complexity (CAA), (b) colour boundary strength (BMS), and (c) luminance boundary strength (BMSL) at 1 m viewing distance and (d) CAA, (e) BMS and (f) BMSL at 5 m viewing distance. Observers are lizard (Li), grey butcherbird (Bb), laughing kookaburra (Kb) and humans (Hu). Error bars are 95% CIs. Different letters above bars within plots indicated pairwise differences.
